# Supplementary material for: Photonics-integrated terahertz transmission lines
Source: Nat Commun. 2025 Jul 30;16:7004. doi: 10.1038/s41467-025-62267-y (PMC12311040; doi:10.1038/s41467-025-62267-y)
Supplement: Supplementary file 1 — Supplementary Information [file 41467_2025_62267_MOESM1_ESM.pdf]

Supplementary information for

Photonics-integrated terahertz transmission lines

Y. Lampert, A Shams-Ansari, A. Gaier, A. Tomasino, X. Cao, L. Magalhaes, S. Rajabali, M. Lončar, I.-C. Benea-Chelmus

Contents

1 Details on TFLN samples and experimental setup **2**

A Geometry and properties of the Lithium Niobate devices . . . 2

B Measurement of the pump group index . . . 2

C Experimental setup for terahertz emission . . . 3

D Experimental setup for terahertz detection . . . 5

2 Theory and modeling of photonics-integrated TFLN emitters **8**

A Theory of terahertz generation inside photonics-integrated transmission lines . . . 8

B Effect of pulse length on THz bandwidth . . . 10

C Estimation of generated terahertz fields confined to the transmission line . . . 10

D Modelling transmission line cavity . . . 12

E Dispersion of THz transmission line . . . 13

F Time-delayed terahertz dipole sources . . . 15

G Farfield analysis . . . 15

3 Further supporting measurements of photonics-integrated TFLN emitters **19**

A Estimation of collection and detection efficiencies . . . 19

B Shot-noise limited detection using electro-optic sampling . . . 19

C Effect of transmission line length for terahertz generation . . . 20

4 Theory photonics-integrated TFLN detectors **22**

A Theory of terahertz detection inside photonics-integrated transmission lines . . . 22

5 Further supporting measurements of photonics-integrated TFLN detectors **26**

A Effect of silicon lens on terahertz detection . . . 26

B Effect of transmission line length on terahertz detection . . . 27

6 Comparison to state of the art **28**

A Power handling of common terahertz materials . . . 28

B Benchmarking of our TFLN emitters to commercial photoconductive antenna . . . 29

C Benchmarking of our TFLN detectors to commercial photoconductive antenna . . . 30

D Overview of key metrics and properties for integrated hybrid terahertz devices . . . 32

1 Details on TFLN samples and experimental setup

A Geometry and properties of the Lithium Niobate devices

Details on the geometry of the measured samples are given in Supplementary Fig. 1 and all dimensions are provided in Supplementary Table 1. These dimensions are used for our CST simulation. The extraordinary refractive index of Lithium Niobate in the telecom range is calculated from [1] and imported to the material properties to determine the effective group refractive index for TE mode inside the X-cut Lithium Niobate rib waveguide. Similarly, the refractive index at THz frequencies is calculated using the double Lorentz model and its coefficients from [2]. Since Lithium Niobate is transparent in the telecom range, its absorption is neglected. In contrast, it is included for our THz simulations.

thickness of the Si substrate	h_{Si}	500 μm
thickness of the SiO_2 layer	h_{SiO_2}	4.7 μm
thickness of Thin-Film $LiNbO_3$ layer	h_{TF}	300 nm
thickness of the transmission line layer	h_{TL}	300 nm
thickness of the antenna layer	h_{ant}	300 nm
height of the $LiNbO_3$ waveguide	h_{wg}	600 nm
thickness of the SiO_2 cladding	$h_{cladding}$	1 μm
narrow width of the $LiNbO_3$ waveguide	$w_{wg,narrow}$	1.5 μm
width of the $LiNbO_3$ waveguide	$w_{wg,wide}$	1.85 μm
width of the SiO_2 cladding	$w_{cladding}$	3.3 μm
width of the antenna	w_{ant}	5 μm
width of the gold layer inside the transmission line	w_{TL}	3.5 μm
width of the gap between the two leads of the transmission line	w_g	3.3 μm
length of the transmission line	l_{TL}	120 μm
length of the antenna	l_{ant}	200 μm

Supplementary Table 1: Dimensions of the simulated and fabricated devices

B Measurement of the pump group index

We measured the transmission spectrum of the $LiNbO_3$ waveguide using the tunable continuous-wave laser (Keysight, 8164A) with a resolution of 0.1 pm. Due to the reflection at the facets of the chip, the modulation of transmission spectrum is observed (Fig. 2), which we attribute to the formation of the Fabry-Perot modes in the waveguide. Taking the difference between local maxima of the transmission, we retrieve the group index using the expression:

$$n_g = \frac{\lambda^2}{2 \cdot \Delta\lambda \cdot L} \tag{1}$$

λ is the wavelength, $\Delta\lambda$ is the difference between two maxima, and L is the length of the waveguide.

Averaging over the range 1549.75-1550.25 nm, we retrieved group index at optical frequency to be $n_g = 2.2503 \pm 0.001$, in line with simulations shown in Fig. 1 of the main text.

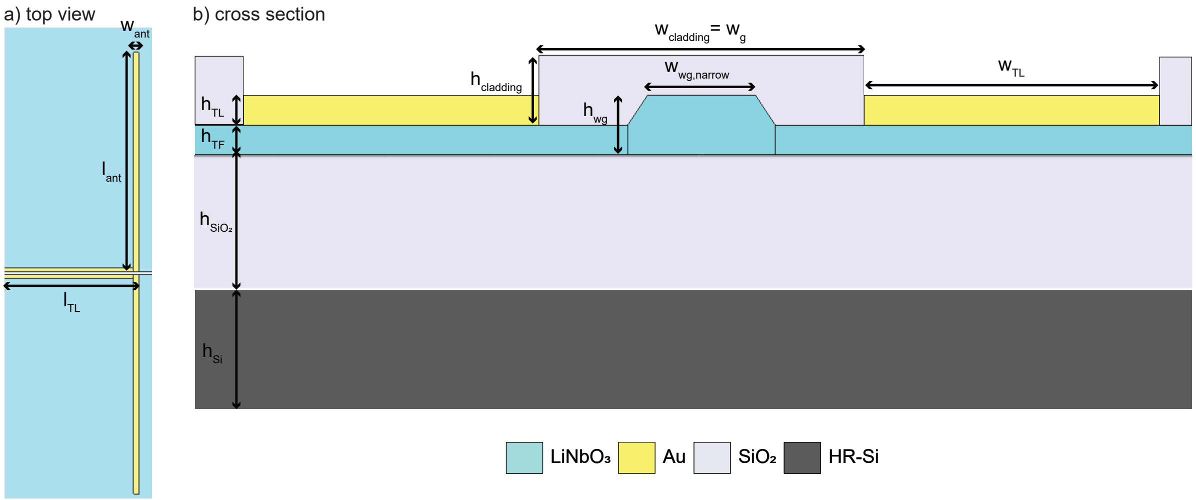

Supplementary Fig. 1: Top view and cross section of the fabricated chip. **a** top view of the device showing the transmission line with length l_{int} terminated by a dipole antenna with length l_{ant} and width w_{ant}. **b** cross section of the transmission line.

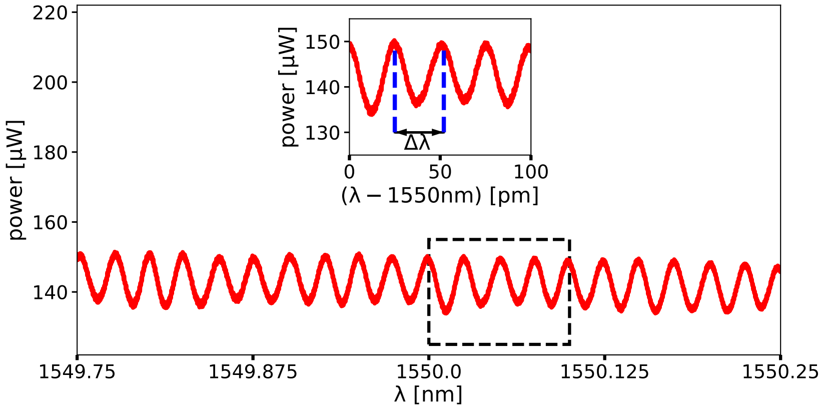

Supplementary Fig. 2: Transmission spectrum of the $LiNbO_3$ waveguide with length L = 21.5mm.

C Experimental setup for terahertz emission

The experimental setup for the characterization of TFLN emitters is depicted in Supplementary Fig. 3. Detection is done with conventional electro-optic sampling [3]. A probe beam at $\lambda_{\text{pr}} = 780$ nm with pulse length of 200 fs is focused at the detection crystal and its polarization is converted into circular at a subsequent quarter wave plate. The two orthogonal polarization components are split using a polarization beam splitter (Wollaston prism) and the difference in optical power is measured using balanced detection, thereby providing common-mode noise rejection. The terahertz beam induces birefrengence at the detection crystal which introduces a slight ellipticity in the otherwise circularly polarised probe beam that eventually leads to intensity imbalance at the balanced detector which is proportional to the incident terahertz electric field. The balanced detector has a bandwidth of 200 MHz which is larger than the 100 MHz repetition rate of the mode locked laser. Therefore, a low-pass with 80 MHz cut-off is used before feeding the voltage signal to the lock-in detector. ZnTe is used for electro-optic sampling of the emitted THz fields and its specifications are provided in Supplementary Table 2 [4] where $\omega_{\text{pr}} = 2\pi \times \frac{c_0}{\lambda_{\text{pr}}}$ is the angular frequency of the probe beam. Both THz and probe are aligned along the y-axis of the lab which is parallel to the $[1\bar{1}0]$ axis of the detection crystals, enabling use of the nonlinear coefficient r_{41} to detect the terahertz electric field.

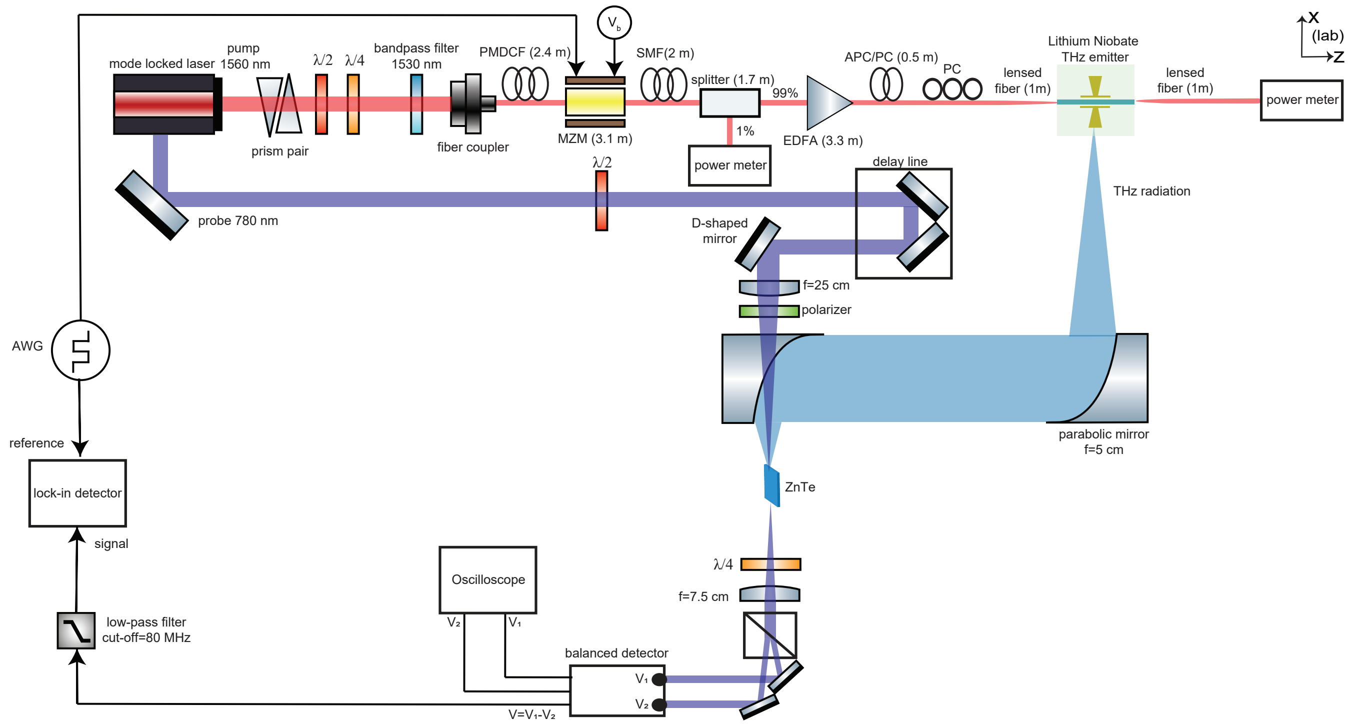

Supplementary Fig. 3: Experimental setup for THz generation. PMDCF: polarization maintaining dispersion compensating fiber, SMF: single mode fiber,MZM: Mach-Zehnder-modulator, EDFA: Erbium-Doped Fiber Amplifier, APC: angle physical contact, PC: physical contact, AWG: arbitrary wave generator.

We apply the following formula to retrieve the THz electric field in absolute values for the Fig. 2, 3 and 4 in the main text [5]

$$\mathrm{E_{det}(t)} = \frac{\mathrm{c_0 \cdot \Delta I}}{\mathrm{I_{pr} \cdot \omega_{pr} \cdot r_{41} \cdot L \cdot n(\omega_{pr})^3}} \tag{2}$$

crystal	thickness L	nonlinear coefficient	crystal orientation	$n(\omega_{THz})$	$n(\omega_{pr})$
ZnTe	1 mm	r_{41}=3.9 pm/V	$[1\bar{1}0]$	3.4	2.87

Supplementary Table 2: Specifications of ZnTe for electro-optic sampling [4]

ΔI is the difference in detected intensity which is induced by the THz field. Experimentally, it is calculated using $\Delta I = \Delta V/G_{RF}$ where ΔV is the voltage difference between the two photodiodes at the RF output of the balanced detector and G_{RF} is the conversion gain [V/W] of the photodetector. P_{pr} is the total probe intensity. It is determined by reading out the voltage from the DC monitor of each photodiode and accounting for the DC signal gain G. This leads to the formula $I_{pr} = V_1/G + V_2/G$. To achieve balanced detection, the polarization of the probe is adjusted such that $V_1 = V_2$. The crystal response can be summarized into a function $F(\omega_{pr}, \omega_{THz})$ which contains absorption, phase matching at a fixed crystal length L, strength of the nonlinearity and the Nyquist limit determined by the pulse length of the probe, providing a mean to compare the two crystals [3, 6]:

$$\mathrm{F(\omega_{pr}, \omega_{THz}) = A_{opt}(\omega_{THz}) \cdot r_{41} \cdot \Delta\phi(\omega_{pr}, \omega_{THz})} \tag{3}$$

$A_{opt}(\omega_{THz})$ is the autocorrelation of the probe pulse which is estimated to have FWHM pulse length of 200 fs.

$$\mathrm{A_{opt}(\omega_{THz})} = \int_{-\infty}^{\infty} \mathrm{E_{pr}(\omega'_{pr} - \omega_{pr})^* E_{pr}(\omega'_{pr} - \omega_{pr} - \omega_{THz}) d\omega'_{pr}} \tag{4}$$

$\Delta\phi(\omega_{\text{pr}}, \omega_{\text{THz}})$ is the phase matching function of the used crystal

$$\Delta\phi(\omega_{\text{Pr}}, \omega_{\text{THz}}) = \frac{e^{i\Delta kL} - 1}{i\Delta k} \tag{5}$$

$$\Delta k = \frac{\Big(\omega_{\text{pr}} \cdot n(\omega_{\text{pr}}) + \omega_{\text{THz}} \cdot (n(\omega_{\text{THz}}) + i\kappa(\omega_{\text{THz}})) - (\omega_{\text{pr}} + \omega_{\text{THz}}) \cdot n(\omega_{\text{pr}} + \omega_{\text{THz}})\Big)}{c_0} \tag{6}$$

Both absorption and dispersion have been included. The results of our calculations are shown for a ZnTe crystal of length L = 1 mm in Supplementary Fig. 4.

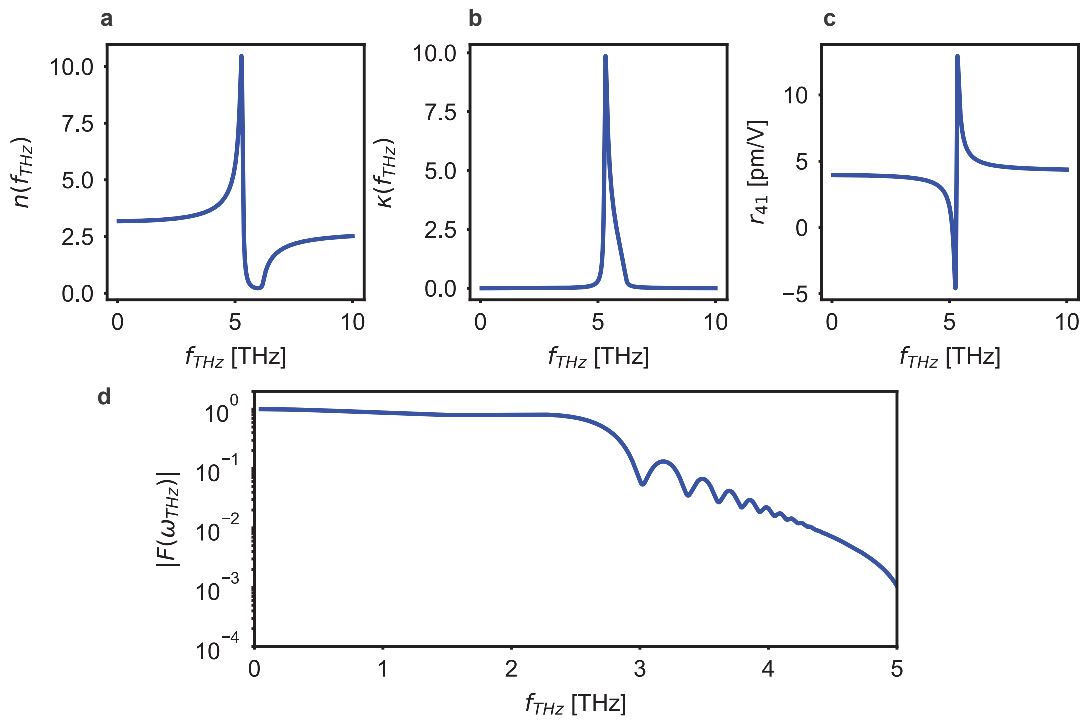

Supplementary Fig. 4: Properties of a 1 mm ZnTe as a detection crystal. **a** Real part of the refractive index. **b** Imaginary part of the refractive index. **c** Nonlinear electro-optic coefficient r_{41}. **d** Calculated response using equation 3 with probe wavelength of 780 nm.

To generate terahertz pulses, we use a telecom pump at 1550 nm which we characterize at the input of the chip using an autocorrelator. A hyperbolic sech fit provides a pulse length of 60 fs, illustrated in Supplementary Fig. 5 a. The optical spectrum at input of the chip is measured using an optical spectrum analyzer as shown in Supplementary Fig. 5 b. Spectral broadening is clearly observed after propagation in fiber and the spectrum at the chip's input spans over 100 nm. To confirm that broad spectra can be coupled into the Lithium Niobate devices, the transmission is characterized and the performance is compared to grating couplers.

Supplementary Fig. 5 c shows the total transmission for the two facets as function of wavelength using grating couplers and edge coupling. In the edge coupling we implement, mode-matching between the waveguide and the lensed optical fiber is enhanced by adiabatically tapering the rib $LiNbO_3$ waveguide from a width of 1500 nm to 800 nm at the edge of the chip over a length of 3 mm. Edge-coupling allows for a broadband operation of the device accommodating for the entire bandwidth of femtosecond pulses.

D Experimental setup for terahertz detection

The measurements shown in Fig. 5 in the main text have been conducted with the setup shown in Supplementary Fig. 6. As an emitter, we use a commercial PCA (iPCA-21-05-1000-800-h from Batop GmbH) for all measurements

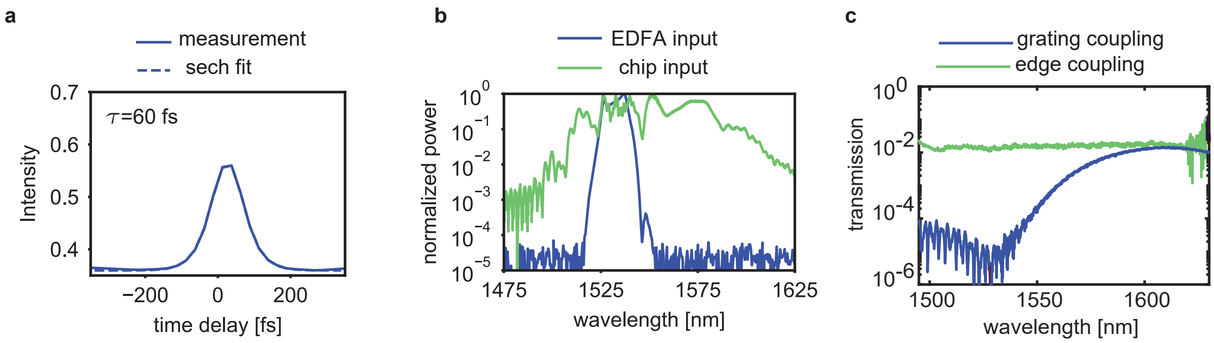

Supplementary Fig. 5: Characterization of the optical pump. **a** pulse intensity measurement using autocorrelation. **b** optical spectrum at input of the EDFA and input of the THz emitter. **c** Total transmission of the Lithium Niobate waveguide (for the two facets) using edge coupling compared to grating couplers.

with 120 mW pump power at 780 nm wavelength and 10 V square wave modulated at 30 kHz. Half and quarter waveplates are rotated to align the polarization of the 1560 nm probe to the TE polarization of the TFLN waveguide (z-axis of the crystal). As in the case of generation, the terahertz wave is aligned to the z-axis to access the nonlinear coefficient $\chi^{(2)}_{333}$. By using a highly nonlinear fiber (HNLF) with length 25 cm and an in-coupled probe power value around 15 mW, we achieved pulse compression down to 60 fs. This probe is then coupled to our TFLN chip through edge coupling. Finally, 13.7 μW of optical power is coupled out and acquired by a photodiode whose voltage readout is connected to a lock-in amplifier synchronized to the bias modulation frequency.

To provide accurate numbers for the sensitivity of our TFLN chips, it is important to calibrate the terahertz pulse. To achieve this, we use a gallium phosphide crystal to perform free-space electro-optic sampling. We choose the crystal to have a thickness of L = 200 μm to ensure a flat frequency response up to 5 THz [6]. This allows us to characterize the temporal shape and frequency content of the input pulse with minimal distortions that could originate from the detector. We replace the fiber system by free space components to focus the probe beam and terahertz radiation on the GaP crystal and measure the birefringence that is induced by the terahertz radiation in the nonlinear crystal via ellipsometry measurements. From the modulation of the optical beam ΔI, we compute the terahertz electric field

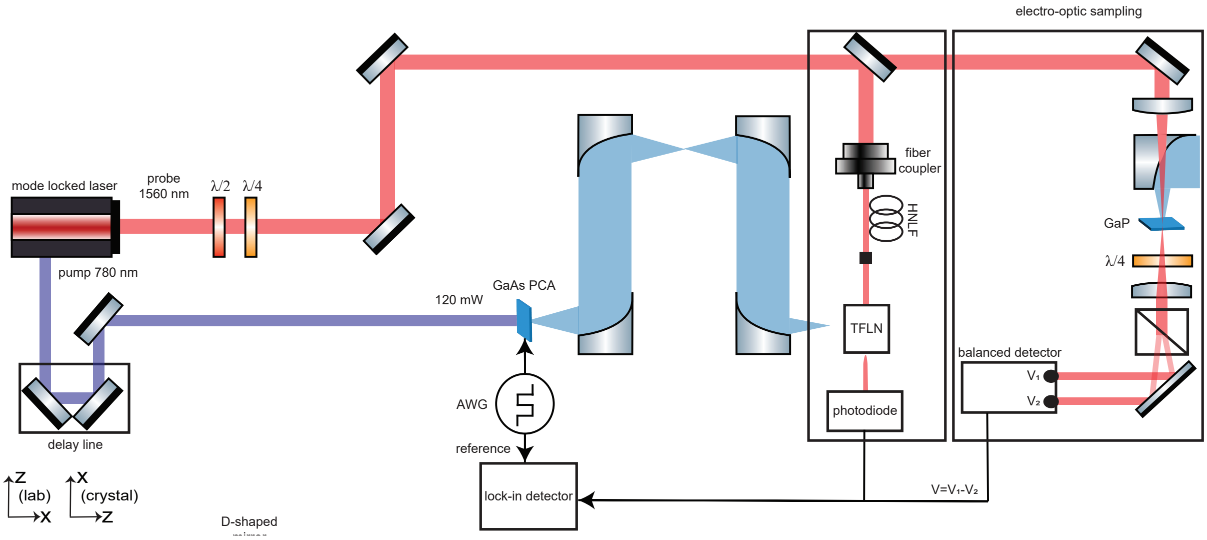

Supplementary Fig. 6: Experimental setup for THz detection in TFLN. AWG: arbitrary wave generator. HNLF: highly nonlinear fiber.

inside the GaP detection crystal using equation:

$$E_{det}(t) = \frac{c_0 \cdot \Delta I}{I_{pr} \cdot \omega_{pr} \cdot r_{41} \cdot L \cdot n(\omega_{pr})^3} \quad (7)$$

and the corresponding refractive indices and nonlinear coefficients for GaP summarised in Supplementary Table 3. To calculate the terahertz field before the chip, we account for the transmission at the crystal/air interface:

$$E_{THz,input}(t) = E_{det}(t) \cdot \frac{n(\omega_{pr}) + 1}{n(\omega_{pr}) - 1} \quad (8)$$

The retrieved input terahertz electric field is shown in absolute units in Fig.5 b of the main text. We provide the measurement configurations in Supplementary Table 4

crystal	thickness L	nonlinear coefficient	crystal orientation	$n(\omega_{THz})$	$n(\omega_{pr})$
GaP	0.2 mm	r_{41}=0.87 pm/V	$[1\bar{1}0]$	3.2	3.05

Supplementary Table 3: Specifications of GaP for electro-optic sampling.

	TFLN	EOS
detector	MZM with dipole antenna	GaP crystal
detector details	$l_{ant} = 200\mu m, l_{TL} = 1mm$	L=0.2 mm r_{41}=0.87 pm/V
emitter	GaAs PCA (iPCA-21-05-1000-800-h from Batop GmbH)	
signal on emitter	square wave with 10 V peak to peak	
modulation frequency	15.1 kHz	30.1 kHz
pump power	120 mW	
pulse lengths	60 fs for pump and probe	
probe setup	fiber coupled: 25 cm HNLF and 15 cm bare fiber	free-space
power before detector	300 μW on chip	5 mW at crystal
power after detector	13.7 μW	1 mW
readout	photodiode	balanced detection
delay line step size	25 fs	
integration time per step	100 ms	300 ms

Supplementary Table 4: setup configurations for comparing different detectors

2 Theory and modeling of photonics-integrated TFLN emitters

A Theory of terahertz generation inside photonics-integrated transmission lines

In this section, we derive the expressions for the THz electric field generated during the propagation of the femtosecond pulse in optical waveguide. Here, we follow the same procedure, which was developed in [7]. We consider the femtosecond pulses to propagate in the optical waveguide along z lab axis. Their electric field is linearly polarised (x component) and given by:

$$E_p(x,y,z,t) = \frac{1}{2}E_0\varepsilon(z,t)g_{opt}(x,y)e^{i(\omega_p t - k_p z)} + c.c. \tag{9}$$

where ω_p is center carrying frequency, $k_p = 2\pi n_p/\lambda_p$ is the wave vector, $g_{opt}(x,y)$ is the optical mode profile, normalized such that $\iint_{(x,y)} |g_{opt}(x,y)|^2 dxdy = S_{eff}$, where S_{eff} is the effective mode area, $\varepsilon(z,t)$ is an envelope slowly varying during the propagation, and E_0 is the pump pulse amplitude. The normalization is such, that the $\int_{-\infty}^{\infty}\iint_{(x,y)} 1/2\varepsilon_0 c_0 n_p |E_p(x,y,z=0,t)|^2 dxdydt = J_{opt}$, where J_{opt} is the pulse energy, and c_0 is the speed of light in vacuum. The terahertz wave is generated via difference frequency generation between frequency components belonging to the pulse. The evolution of the THz electric field is described by the non-linear wave equation:

$$\left[\nabla^2 - \frac{1}{c_0^2}\frac{\partial^2}{\partial t^2}\right]E_{THz}(x,y,z,t) = \frac{1}{\varepsilon_0 c_0^2}\frac{\partial^2}{\partial t^2}\left(P^{L}_{THz}(x,y,z,t) + P^{NL}_{THz}(x,y,z,t)\right) \tag{10}$$

where ε_0 is the vacuum permittivity. The linear and non-linear polarizations, $P^{L}_{THz}(x,y,z,t)$ and $P^{NL}_{THz}(x,y,z,t)$ take the form of:

$$P^{L}_{THz}(x,y,z,t) = \varepsilon_0\int_{-\infty}^{t}\chi^{(1)}(t-t')E_{THz}(x,y,z,t')dt' \tag{11}$$

$$P^{NL}_{THz}(x,y,z,t) = \varepsilon_0\chi^{(2)}|E_p(x,y,z,t)|^2 \tag{12}$$

where $\chi^{(1)}$ is the complex linear susceptibility, and $\chi^{(2)} \approx 360 pm/V$ is the second-order susceptibility of $LiNbO_3$ at microwave and terahertz frequencies (below the phonon lines) [8, 9, 10]. Taking the Fourier transformation of 10, taking into account the relation between linear polarization and electric field $\tilde{P}^{L}_{THz}(x,y,z,t) = \varepsilon_0\chi^{(1)}(\omega_{THz})\tilde{E}_{THz}(x,y,z,\omega_{THz})$, where tilde stands for the Fourier transforms, we obtain the following equation:

$$\left[\nabla^2 + \frac{\omega_{THz}^2}{c_0^2}(n(\omega_{THz}) + i\kappa(\omega_{THz}))^2\right]\tilde{E}_{THz}(x,y,z,\omega_{THz}) = \frac{\chi^{(2)}}{c_0^2}\int_{-\infty}^{+\infty}\frac{\partial^2}{\partial t^2}(|E_p(x,y,z,t)|^2)\cdot e^{-i\omega_{THz}t}dt \tag{13}$$

where $n(\omega_{THz}) + i\kappa(\omega_{THz}) = \sqrt{1+\chi^{(1)}(\omega_{THz})}$ is the effective complex refractive index at THz frequency. The evolution of the pump envelope is described by:

$$\varepsilon(z,t) = \frac{1}{2\pi}\int_{-\infty}^{+\infty}\tilde{\varepsilon}(0,\omega_{THz})e^{i\omega_{THz}(t-\frac{z}{c_0}n_g)}d\omega_{THz} \tag{14}$$

$\tilde{\varepsilon}(0,\omega_{THz})$ is Fourier transform of the pulse envelope at the beginning of the transmission line, n_g is the group index at central pump frequency. We assume the pulse profile at the boundary z=0 has the form of:

$$\varepsilon(0,t) = \mathrm{sech}(t/\tau) \tag{15}$$

where $\tau \approx \frac{FWHM}{1.76}$ is the pulse duration, FWHM is the full width at half maximum determined with pulse intensity. We assume that the transmission line supports one mode, the spatial profile of which does not depend on the THz frequency so that we can express the THz electric field as

$$\tilde{E}_{THz}(x,y,z,\omega_{THz}) = \tilde{E}^{(amp)}_{THz}(z,\omega_{THz})\cdot e^{-i\frac{\omega_{THz}}{c_0}n(\omega_{THz})z}\cdot g_{THz}(x,y). \tag{16}$$

where $g_{THz}(x,y)$ is the THz mode profile, normalized such that $\iint_{(x,y)} |g_{THz}(x,y)|^2 dxdy = S_{THz,eff}$, where $S_{THz,eff}$ is the effective mode area of THz mode. By substituting Eq. 14 into Eq. 13:

$$\left[\nabla^2 + \frac{\omega_{THz}^2}{c_0^2}(n(\omega_{THz}) + i\kappa(\omega_{THz}))^2\right] \tilde{E}_{THz}^{(amp)}(z,\omega_{THz}) \cdot e^{-i\frac{\omega_{THz}}{c_0}n(\omega_{THz})z} \cdot g_{THz}(x,y) = \frac{-\pi\chi^{(2)}E_0^2\omega_{THz}^3\tau^2}{2c_0^2 \sinh(\pi\omega_{THz}\tau/2)} e^{-i\frac{\omega_{THz}}{c_0}n_g z} \cdot g_{opt}^2(x,y) \tag{17}$$

To solve this equation, we apply the following simplifications: first, we neglect the derivatives with respect to x and y axis, second, we can multiply both parts of the equation by $g_{THz}(x,y)$ and integrate over x and y coordinates. This procedure allows us to do the analysis of 1D case, where we describe only propagation along z axis. We can introduce the overlap factor between THz and optical modes, which impacts the THz generation efficiency at a given mode:

$$\Gamma_{OR} = \frac{\iint_{(x,y)} g_{opt}^2(x,y) g_{THz}(x,y) dxdy}{\iint_{(x,y)} |g_{THz}(x,y)|^2 dxdy} \tag{18}$$

Next, we can rewrite Eq. 17 as

$$\left[\frac{\partial^2}{\partial z^2} + \frac{\omega_{THz}^2}{c_0^2}(n(\omega_{THz}) + i\kappa(\omega_{THz}))^2\right] \tilde{E}_{THz}^{(amp)}(z,\omega_{THz}) \cdot e^{-i\frac{\omega_{THz}}{c_0}n(\omega_{THz})z} = \frac{-\pi\chi^{(2)}E_0^2\omega_{THz}^3\tau^2}{2c_0^2\sinh(\pi\omega_{THz}\tau/2)} e^{-i\frac{\omega_{THz}}{c_0}n_g z} \cdot \Gamma_{OR} \tag{19}$$

We can now simplify Eq. 19 by applying the slowly-varying amplitude approximation to the THz wave, thus neglecting the second derivative with respect to the z-coordinates. Moreover, we can assume that the THz absorption is weak $|n(\omega_{THz})| \gg |\kappa(\omega_{THz})|$, so that $(n(\omega_{THz}) + i\kappa(\omega_{THz}))^2 \approx n^2(\omega_{THz}) + 2i\kappa(\omega_{THz})n(\omega_{THz})$. We can now rearrange the term on the left side of the equation as:

$$\left[\frac{\partial^2}{\partial z^2} + \frac{\omega_{THz}^2}{c_0^2}(n(\omega_{THz}) + i\kappa(\omega_{THz}))^2\right] \tilde{E}_{THz}^{(amp)} \cdot e^{-i\frac{\omega_{THz}}{c_0}n(\omega_{THz})z} \approx \frac{\partial \tilde{E}_{THz}^{(amp)}}{\partial z} \cdot e^{-i\frac{\omega_{THz}}{c_0}n(\omega_{THz})z} \cdot \left(-2i\frac{\omega_{THz}}{c_0}n(\omega_{THz})\right) + 2i\kappa(\omega_{THz})n(\omega_{THz})\frac{\omega_{THz}^2}{c_0^2}\tilde{E}_{THz}^{(amp)} \cdot e^{-i\frac{\omega_{THz}}{c_0}n(\omega_{THz})z} \tag{20}$$

Introducing the loss coefficient $\alpha(\omega_{THz}) = \frac{2\omega_{THz}\kappa(\omega_{THz})}{c_0}$ and substituting this into Eq. 19 gives the equation for the evolution of the THz field:

$$\frac{\partial \tilde{E}_{THz}^{(amp)}}{\partial z} = \frac{-i\pi\chi^{(2)}E_0^2\omega_{THz}^2\tau^2}{4c_0 \cdot n(\omega_{THz}) \sinh(\pi\omega_{THz}\tau/2)} e^{-i\Delta kz} \cdot \Gamma_{OR} - \frac{\alpha(\omega_{THz})}{2}\tilde{E}_{THz}^{(amp)} \tag{21}$$

where $\Delta k = \frac{\omega_{THz}}{c_0}(n_g - n(\omega_{THz}))$ is the wave vectors mismatch. Considering the boundary condition

$$\tilde{E}_{THz}^{(amp)}(z=0,\omega_{THz}) = 0 \tag{22}$$

We find the solution of the equation for propagation in our transmission line with length l_{TL} and refractive index $n(\omega_{THz}) = n_{TL}(\omega_{THz})$:

$$\tilde{E}_{THz}^{(amp)}(l_{TL},\omega_{THz}) = \frac{i\pi\chi^{(2)}E_0^2\omega_{THz}^2\tau^2 l_{TL}\Gamma_{OR}}{4c_0 n_{TL}(\omega_{THz}) \sinh(\pi\omega_{THz}\tau/2)} G_{TL}(\omega_{THz}) \tag{23}$$

where we introduce the phase-matching function $G_{TL}(\omega_{THz}) = \frac{e^{-i\Delta k l_{TL}} - e^{-\alpha l_{TL}/2}}{i\Delta k l_{TL} - \alpha l_{TL}/2}$ that quantifies the generation efficiency. One may introduce the effective length $l_{eff} = l_{TL} \cdot G_{TL}(\omega_{THz})$, which describes the length on which the generation of radiation at a given frequency happens. In order to calculate the time trace of the THz pulse, we perform the inverse Fourier transform:

$$E_{THz}(l_{TL},t) = \frac{1}{2\pi}\int_{\omega_{THz}} \tilde{E}_{THz}^{(amp)}(l_{TL},\omega_{THz}) \cdot e^{i\omega_{THz}\left(t - \frac{l_{TL}n_{TL}(\omega_{THz})}{c_0}\right)} d\omega_{THz} \tag{24}$$

And, finally, we can introduce the THz energy pulse J_{THz} as:

$$J_{THz} = \int_{-\infty}^{\infty} \iint_{(x,y)} \frac{1}{2}\varepsilon_0 c_0 n_{eff} |E_{THz}(l_{TL}, t)|^2 \cdot |g_{THz}(x, y)|^2 dxdydt \tag{25}$$

We note that, as discussed in [7], these expressions are valid under the following assumptions: (i) the conversion efficiency $\eta = \frac{J_{THz}}{J_{opt}} \ll \frac{\Delta\omega_p}{\omega_p}$, where $\Delta\omega_p \approx \frac{1.12}{FWHM}$ is the pump pulse bandwidth, and ω_p is pump frequency. In our case, $\frac{\Delta\omega_p}{\omega_p} \approx 5.1 \cdot 10^{-2}$, and as we show, it is much above the generation efficiencies of our device; (ii) the $\chi^{(3)}$-based effects, such as self-phase modulation, are negligible for the optical pump. Nonlinear phase, associated with the Kerr-nonlinearity, is negligible and can be estimated as $\delta\varphi \approx n_2 \cdot I_{peak} \cdot k_p l_{TL} \approx n_2 \frac{J_{opt}}{\tau S_{eff}} \frac{2\pi}{\lambda_p} l_{TL} \approx 10^{-3}$, where I_{peak} is the peak intensity of the pump pulse, k_p is the wave vector, and $n_2 \approx 10^{-19} m^2/W$ is the nonlinear index of $LiNbO_3$ [11].

B Effect of pulse length on THz bandwidth

The scaling of the terahertz field amplitude with pulse length and terahertz frequency can be calculated using equation 23 assuming perfect phase matching:

$$E_{THz} \propto J_{opt} \frac{\omega_{THz}^2 \cdot \tau \cdot l_{TL}}{\sinh(\pi\omega_{THz}\frac{\tau}{2})} \tag{26}$$

The power is given by $|E_{THz}|^2$ which is shown in supplementary Fig. 7 **a**. We show that longer pulses lead to significant drop in efficiency at higher terahertz frequencies. We can estimate the pulse length required to efficiently generate at a desired terahertz frequency by looking at the 3-dB power drop compared to the case of 1 fs (intersection with dashed line in supplementary Fig. 7 **b**). These values are provided in Fig. 1 d right y-axis of the main text.

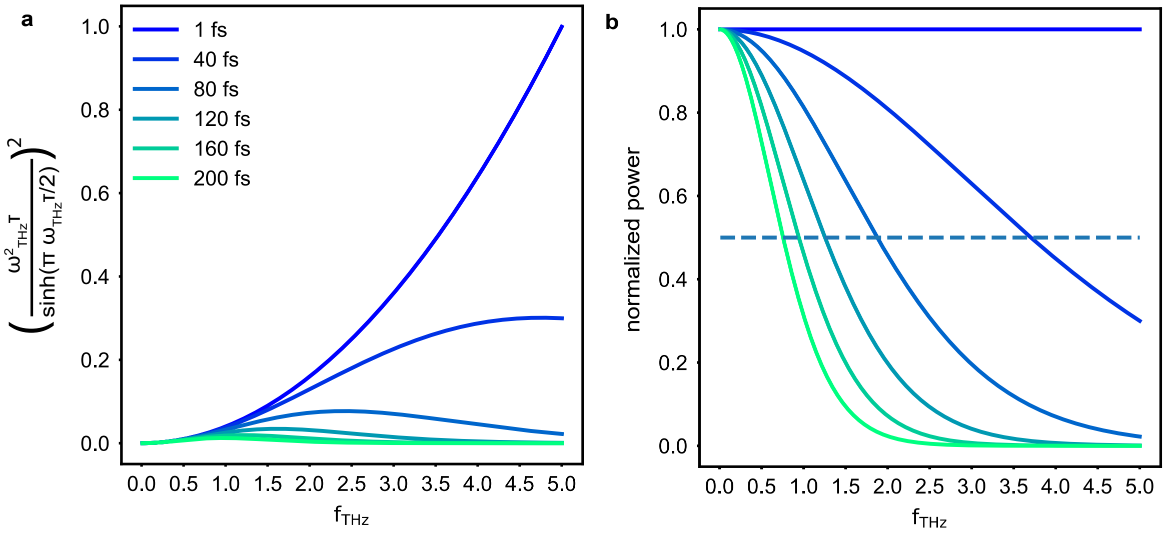

Supplementary Fig. 7: a Power scaling from equation 26 assuming constant pump energy for different pulse lengths. **b** Power values normalized to the case of shortest pulse length $\tau = 1$ fs. Dashed highlights the 3-dB drop in normalized power

C Estimation of generated terahertz fields confined to the transmission line

Using the derivation of section 2 A, we investigate the impact of losses and phase matching onto the generated terahertz field amplitude at the end of a transmission line of arbitrary length. Towards this end, we first simulate the losses, overlap and refractive index for various dimensions gap widths w_g, shown in Supplementary Fig. 9 using CST Studio. We find that an index between $n_{TL} = 2.2 - 2.4$ can be engineered by changing the gap width, and that this influences the losses and the overlap quite significantly. First, we calculate the radiative and absorption losses, and the results are shown in Supplementary Fig. 8. To do this, we perform two simulations: one with lossy materials

(both gold and $LiNbO_3$) and the second simulation with lossless materials. In the first case, the losses come from both radiation and absorption, while in the second case, losses come only from radiation. We launch the propagation over the transmission length $l_{TL} = 100\ \mu m$ and extracted the transmission T as the function of frequency. From this simulation we compute the loss coefficient as follows:

$$\alpha(f_{THz}) = -\frac{1}{l_{TL}}\ln(T(f_{THz})) \tag{27}$$

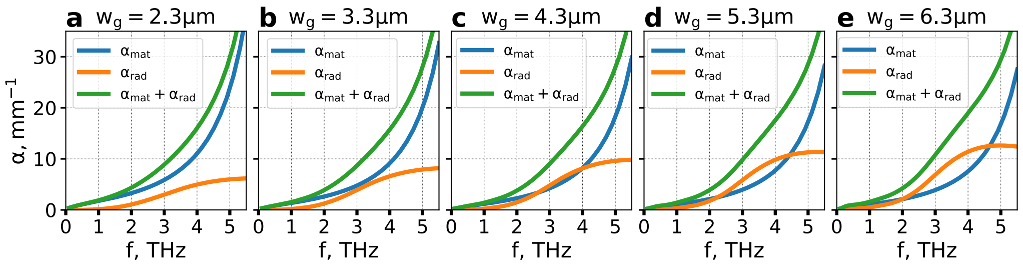

Supplementary Fig. 8: Radiative and absorption losses of the transmission line Radiative loss coefficient α_{rad} and absorption coefficient α_{mat} for the gaps $w_g = 2.3\ \mu m$ (**a**), $w_g = 3.3\ \mu m$ (**b**),$w_g = 4.3\ \mu m$ (**c**), $w_g = 5.3\ \mu m$ (**d**), and $w_g = 6.3\ \mu m$ (**e**).

The difference between the total loss coefficient $\alpha = \alpha_{mat} + \alpha_{rad}$ and radiative loss coefficient α_{rad} gives the absorption coefficient α_{mat}, which is plotted for various w_g in Supplementary Fig. 8 a-e. One may observe that the radiative losses are increasing for larger gap width, while the absorption losses are decreasing, since the THz mode spatial overlap with the absorbing materials ($LiNbO_3$) is decreasing.

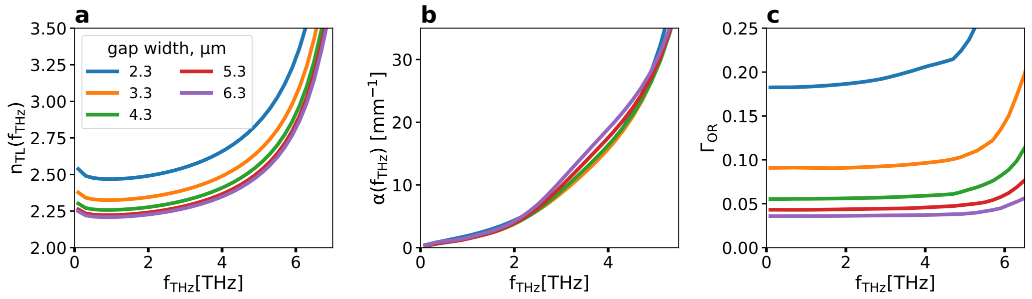

Supplementary Fig. 9: Properties of the transmission lines for various gap widths as a function of frequency **a** Effective index of the transmission line n_{TL}. **b** Absorption losses inside the transmission line. **c** Overlap integral as defined in section A.

Accounting for all of these parameters, we compute the phase matching function $G(\omega_{THz})$ while remarking that, generally, the terahertz field amplitude is dependent as

$$\tilde{E}^{(amp)}_{THz}(l_{TL}, \omega_{THz}) \sim \chi^{(2)} E_0^2 \omega_{THz}^2 l_{TL} \Gamma_{OR} G_{TL}(\omega_{THz}) \tag{28}$$

since $n_{TL}(\omega_{THz})$ is rather flat with frequency. We note that a long transmission line only practical if the total product is larger than the one for a short interaction length. To get an intuition for this, we plot in Supplementary Fig. 10 the phase matching function for varying gap widths and varying transmission line lengths. In general, we find that a transmission line of $l_{TL} = 200\ \mu m$ can provide proper phase matching for frequencies surpassing 3 THz for widths above $w_g = 3\ \mu m$ and that a transmission line of length $l_{TL} = 1$ mm is adequate at frequencies below 1 THz.

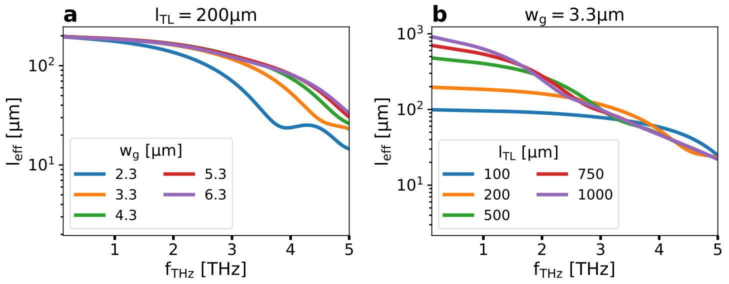

Supplementary Fig. 10: Effective length. **a** For a fixed transmission line length of $l_{TL} = 200\ \mu m$ and with varying transmission line width. **b** For a varying transmission line length and with fixed transmission line width of $w_g = 3.3\ \mu m$, as in our experiments.

The terahertz electric field at the end of a transmission line of given length $L = l_{TL}$ is evaluated using equations 23-24 and the results are presented in Supplementary Fig. 11. For this calculations, we choose the experimental parameters of the optical pump pulse: for a pump pulse energy of $J_{opt} = 1$ pJ and pulse length of $\tau \approx \frac{FWHM}{1.76} \approx 60$ fs. First, using equation 23 we analyzed the generated spectra at the end of the transmission line which are shown in Fig. 11 a and Fig. 11 b. As expected, for longer propagation lengths, the spectrum becomes narrower due to the both absorption and phase-matching conditions. Also, varying the gap between two metallic strips of the transmission line, we show, that the biggest amplitude and the largest bandwidth is achieved for the smallest gap. This is due to the better overlap between optical and terahertz modes and phase-matching conditions. Next, we evaluated the waveforms, using equation 24 and results are shown in subplots Fig. 11 c and Fig. 11 d. We find exemplary for a transmission line length of $l_{TL} = 200\ \mu m$ that electric fields on the order of $E_{THz} = 1$ kV/cm are generated at the end of the transmission line for different gap widths and transmission line lengths, as it is summarized in Fig. 11 e.

We estimated the efficiency of the THz pulse generation $\frac{J_{THz}}{J_{opt}^2}$ to be $\sim 10^{-4}$/pJ, as shown in Fig. 12. We note, that this value does not depend on pump pulse energy, and it gives a conversion efficiency of $\eta = \frac{J_{THz}}{J_{opt}} \sim 10^{-4}$ for pump pulse energy $J_{opt} = 1$ pJ.

D *Modelling transmission line cavity*

In this section, we model the cavity formed by the transmission line discussed in Fig. 4 in the main text. Towards this end, we rely on the derivation of the generated terahertz field of section A, and on the reflection S_{11} and loss α parameters of Fig. 9 we have determined for the transmission lines.

The total outcoupled field E_{out} is a sum over the various contributions of the terahertz pulses travelling back and forth inside the transmission line, as sketched in Fig. 13 a:

$$E_{out} = E_{out}^{(1)} + E_{out}^{(2)} + E_{out}^{(3)} + ... \tag{29}$$

where we have:

$$E_{out}^{(1)} = t_a \tilde{E}_{THz}^{(amp)} \left(\frac{l_{TL}}{2}, \omega_{THz} \right) e^{-i \frac{\omega_{THz}}{c} n_{TL}(\omega_{THz}) \frac{l_{TL}}{2}} \tag{30}$$

$$E_{out}^{(2)} = t_a \cdot \left(S_{11} \cdot \tilde{E}_{THz}^{(amp)} \left(\frac{l_{TL}}{2}, \omega_{THz} \right) e^{-i \frac{\omega_{THz}}{c} n_{TL}(\omega_{THz}) l_{TL}} e^{-\frac{\alpha(\omega_{THz})}{2} \cdot \frac{l_{TL}}{2}} + r_a S_{11} E_{out}^{(1)} e^{-\frac{\alpha(\omega_{THz}) l_{TL}}{2}} e^{-i \frac{\omega_{THz}}{c} n_{TL}(\omega_{THz}) l_{TL}} \right) \tag{31}$$

$$E_{out}^{(3)} = t_a \cdot S_{11} E_{out}^{(2)} e^{-\frac{\alpha(\omega_{THz}) l_{TL}}{2}} e^{-i \frac{\omega_{THz}}{c} n_{TL}(\omega_{THz}) l_{TL}} \tag{32}$$

$$... \tag{33}$$

$$E_{out}^{(i+1)} = t_a \cdot S_{11} E_{out}^{(i)} e^{-\frac{\alpha(\omega_{THz}) l_{TL}}{2}} e^{-i \frac{\omega_{THz}}{c} n_{TL}(\omega_{THz}) l_{TL}} \tag{34}$$

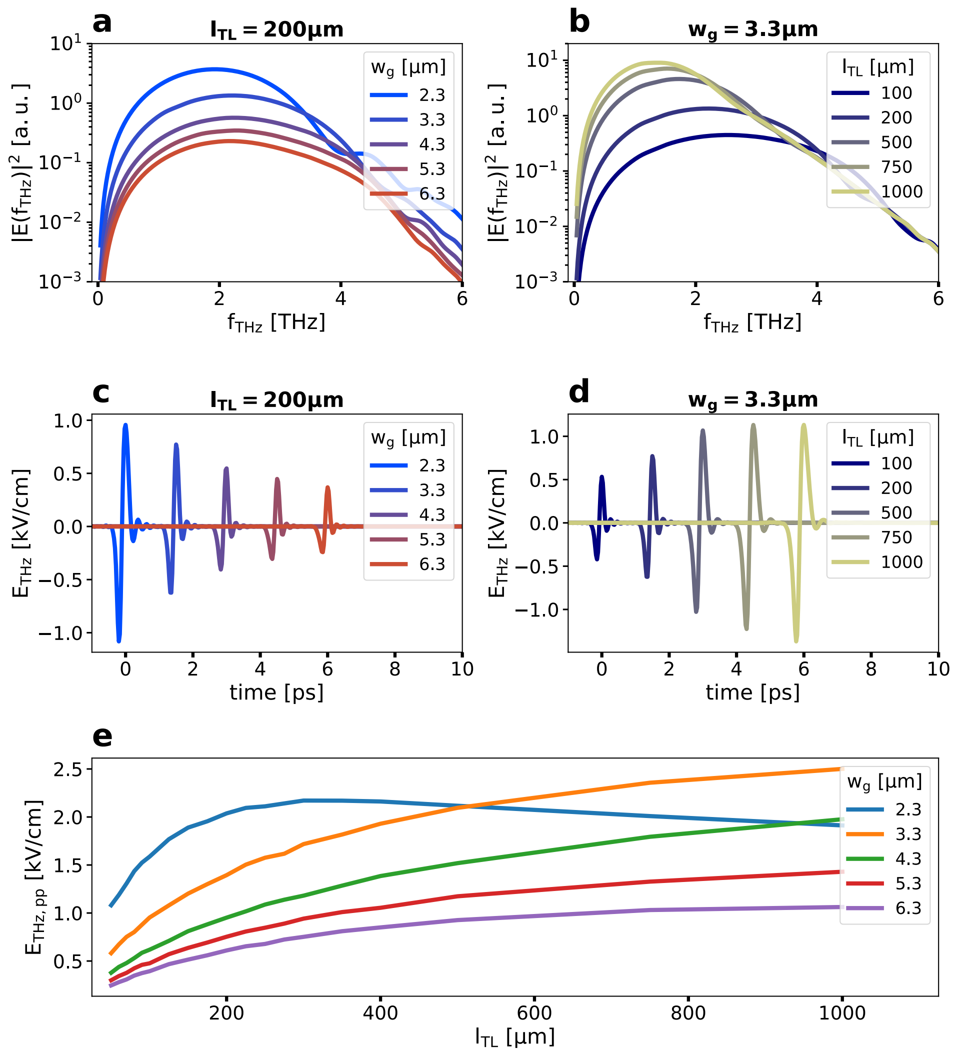

Supplementary Fig. 11: Terahertz electric field at the end of the transmission line as estimated from the analytical model of section A. Calculated spectra **a** and time transients **c** for various gap widths w_g and fixed transmission line length l_{TL}; Calculated spectra **b** and time transients **d** for fixed w_g and various l_{TL}; **e** Peak-to-peak values of the generated THz pulses as a function of length and different waveguide widths.

By assuming the extraction of the antenna to be $t_a = 0.3$ and $t_{TL} = 0.9$, we find a very good agreement between experimental data and modelling, as illustrated in Supplementary Fig. 13 b-c. The time-domain signatures featuring several reflections, and the well-resolved frequency-domain resonances, confirm the emergence of cavity modes through repeated reflections at the open end termination of the transmission line. The constructive interference of these fields locally enhances the terahertz electric field at the resonance condition compared to a single-pass field.

E Dispersion of THz transmission line

Since our design provides a relatively large coherence length, group velocity dispersion (GVD) of the THz mode is calculated. The group index of THz is calculated from the effective refractive index values in the CST simulations. Experimentally, the value is estimated from the free spectral range (FSR) of the cavity modes in Fig. 4 in the main text using the formula

$$\Delta\nu_{FSR} = \frac{c_0}{2n_g \cdot l_{TL}} \tag{35}$$

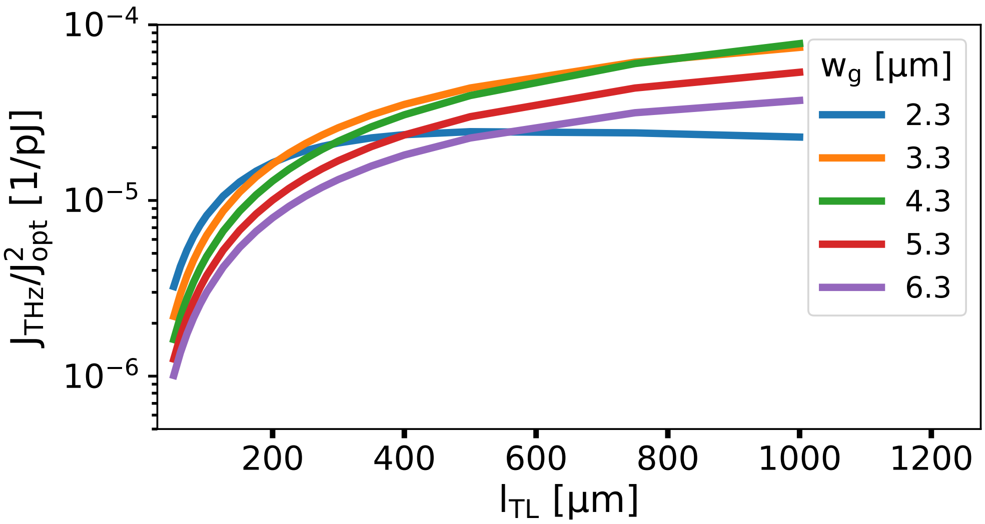

Supplementary Fig. 12: Conversion efficiency of the generation of THz pulses estimated from the analytical model of section A

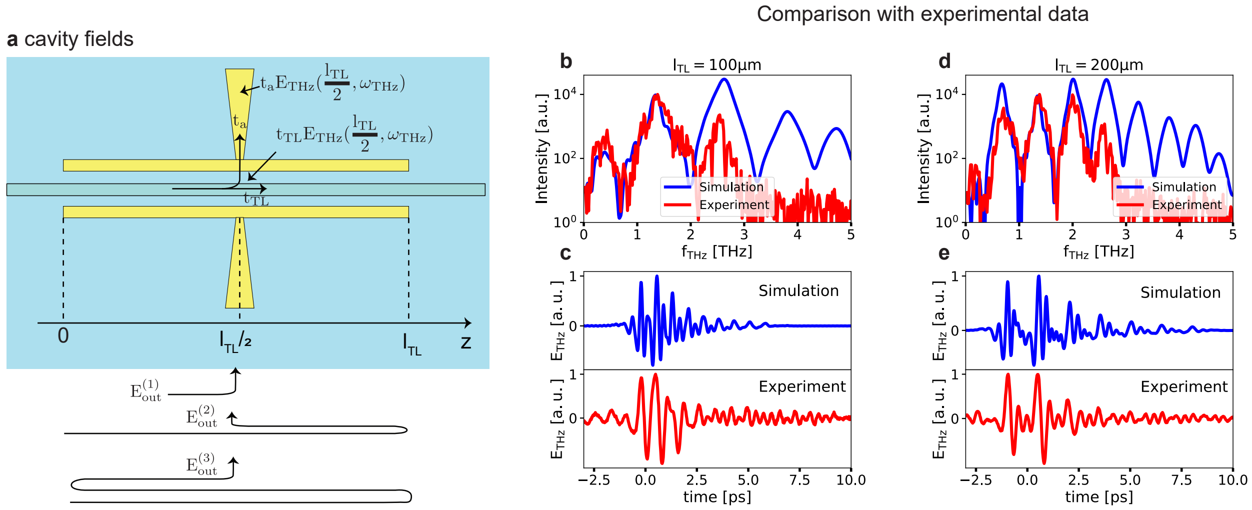

Supplementary Fig. 13: Modelling of the cavity properties. **a** Sketch of the cavity formed by a transmission line with a centrally patterned extracting terahertz antenna. The total outcoupled terahertz electric field is the sum of the outcoupled fields after various round-trips inside the cavity E_{out}. Comparison between experimental data in main text 4e for the transmission line length of **b-c**: 100 μm; **d-e**: 200 μm. Results show good agreement in the number of pulses, as well as in the location of the Fabry-Perot resonances and depth of the dips, confirming the existence of cavity modes.

and it lies in the range 2.1-2.7 which agrees with our simulations. The GVD at a central THz wavelength $\lambda_{THz,0}$ is determined from the simulated refractive index.

$$\mathrm{GVD} = \frac{\lambda^3_{\mathrm{THz},0}}{2\pi c^2}\left(\frac{\partial^2 n}{\partial \lambda^2_{\mathrm{THz}}}\right)_{\lambda_{\mathrm{THz}}=\lambda_{\mathrm{THz},0}} \tag{36}$$

The GVD of our device is compared to the bulk Lithium Niobate (Supplementary Fig. 14 b). Our structure provides around factor 4 decrease in GVD compared to bulk Lithium Niobate. Next, we analyze the effect of the propagation distance l_{TL} and dispersion on the carrier envelope offset ϕ_{ceo} of the THz pulse. This can be calculated using the

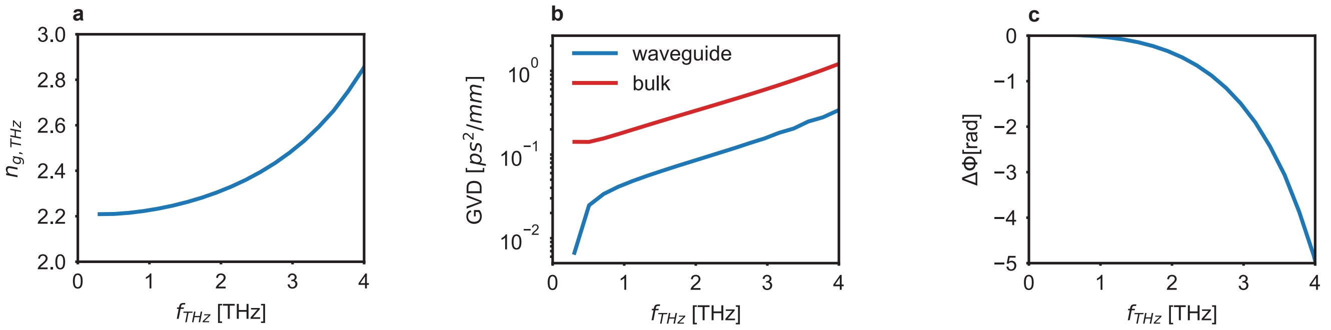

Supplementary Fig. 14: **a** Group index of THz mode. **b** Group velocity dispersion (GVD) of THz radiation in the case of bulk Lithium Niobate and our transmission line mode. **c** Change in phase that originates from propagation in a 120 μm THz transmission line.

formula [12]

$$\phi_{\mathrm{ceo}} = \mathrm{l_{TL}} 2\pi \left(\frac{\partial \mathrm{n}}{\partial \lambda_{\mathrm{THz}}} \right)_{\lambda_{\mathrm{THz}}=\lambda_{\mathrm{THz},0}} \tag{37}$$

We use 120 μm to predict the effect of the transmission line on the THz pulse. As depicted in Supplementary Fig. 14 b, the effect becomes relevant at frequencies above 2 THz. At 3.5 THz, a complete phase flip ($\Delta\phi = 180$ degrees) occurs.

F Time-delayed terahertz dipole sources

To visualize the generation of terahertz radiation from the device in Fig 2 in the main text ($\mathrm{l_{ant}} = 200$ μm and $\mathrm{l_{TL}} = 120\mu$m) in time during propagation of the pump inside the transmission line and during the emission into farfield, 39 subsequent dipole sources are created along the Lithium Niobate waveguide (Supplementary Fig.15 a and b). The excitation of each source m is delayed by $\Delta \mathrm{t_m} = \mathrm{m} \cdot \mathrm{n_g} \cdot \mathrm{d}/\mathrm{c_0}$, where $\mathrm{d} = 10$ μm is the distance between each two subsequent dipole sources. The results of the time domain simulation are shown in the top and side view in 15 c and d with 500 fs time steps. To represent the terahertz generation and guiding, the sources are first excited at position -180 μm where the pump has not yet reached the transmission line. As anticipated, up to the time step 2.5 ps, the generated terahertz is not guided and emission into the substrate is efficient at the Cherenkov angle $\theta_\mathrm{c} = \arcsin \frac{\mathrm{n_g}}{\mathrm{n_{THz}}} \approx 42°$ (highlighted at time 2.5 in d). Once the dipole sources inside the transmission line are excited, the generated terahertz radiation is guided towards the antenna (time 3.5 ps in c). While the real substrate thickness is 500 μm , 100 μm is chosen for the simulation to reduce required memory and computation. The reflection at the substrate-air interface is shown in d at time 5 ps where the diverging terahertz radiation will be collected by the parabolic mirror (shown in the optical setup in Supplementary Fig. 3).

G Farfield analysis

The divergence of the THz field into air as shown in Supplementary Fig. 15 d after time 4.5 ps necessitates analysis of the antenna's radiation pattern at different frequencies to estimate the collection efficiency (Supplementary Fig. 16). Due to the broadband nature of our THz device, the following analytical expression is used to calculate the normalized radiation intensity in the farfield as a function of emission angle θ [13]:

$$\mathrm{U}(\theta, \mathrm{f_{THz}}) = \left[\frac{\cos(\beta_{\mathrm{THz}} \mathrm{l_{ant}} \cos\theta) - \cos(\beta_{\mathrm{THz}} \mathrm{l_{ant}})}{\sin\theta} \right]^2 \tag{38}$$

The total power for each frequency can be then calculated by integrating over the all angles

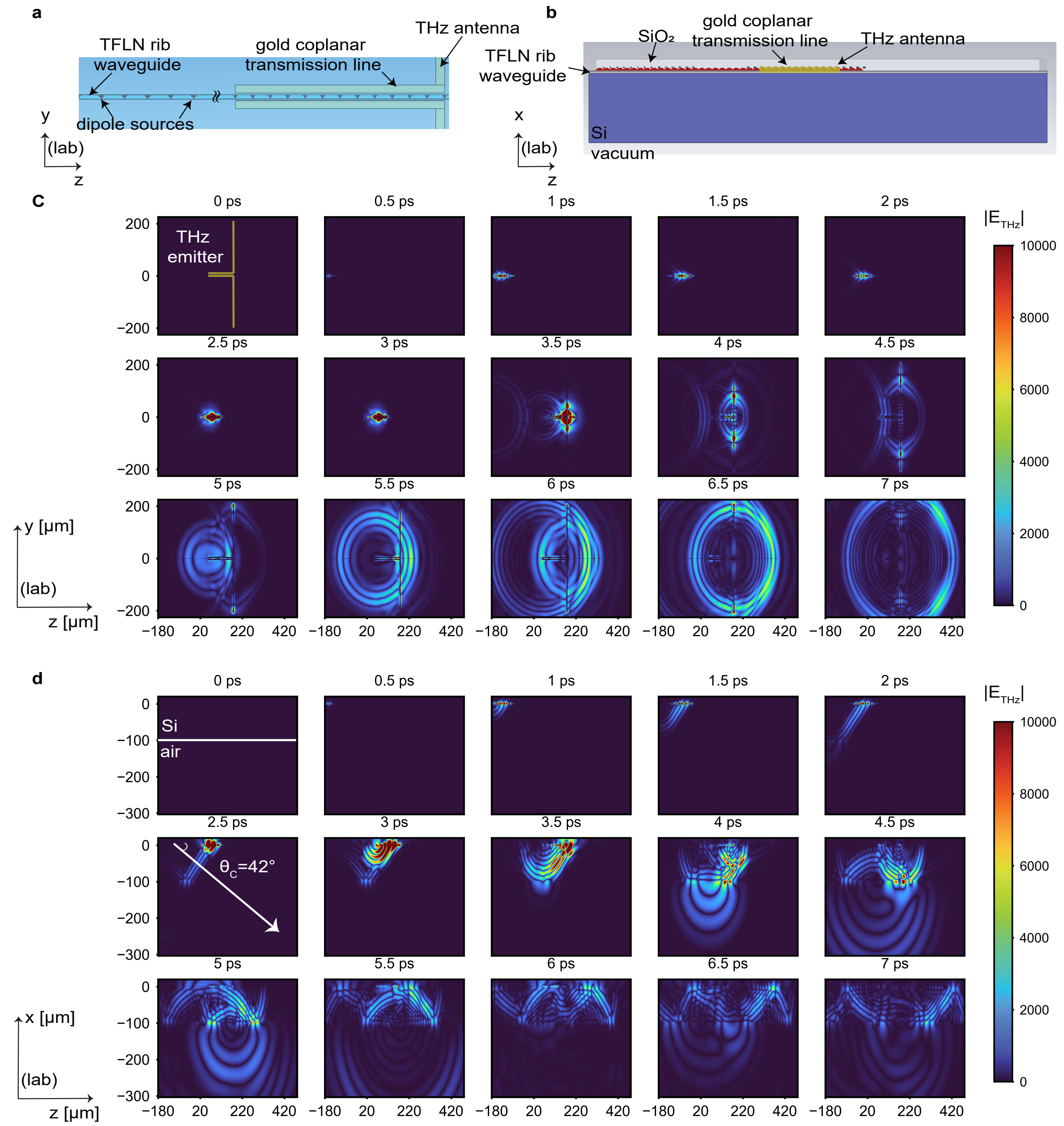

Supplementary Fig. 15: Time-domain CST simulation of delayed THz dipole sources for the dipole antenna in Fig. 2 in the main text. Simulated structure in CST viewed from the top (**a**) and from the side (**b**). **c** top view of the generated $|E_{THz}|$ from the dipole sources with 500 fs time steps. **d** side view of the THz field showing: Cherenkov radiation up to 2.5 ps, confinement of the THz in the transmission line, emission from the antenna into the substrate, reflection at the substrate-air interface and emission into the farfield.

$$\mathrm{P_{total}(f_{THz})} = \int_0^\pi \int_0^{2\pi} \mathrm{U}(\theta, \mathrm{f_{THz}}) \sin\theta \mathrm{d}\phi \mathrm{d}\theta \tag{39}$$

Next, the collection angle α_{coll} is determined from the specifications of the used parabolic mirror:

$$\alpha_{\mathrm{coll}} = \arctan\left(\mathrm{a/RFL}\right) \tag{40}$$

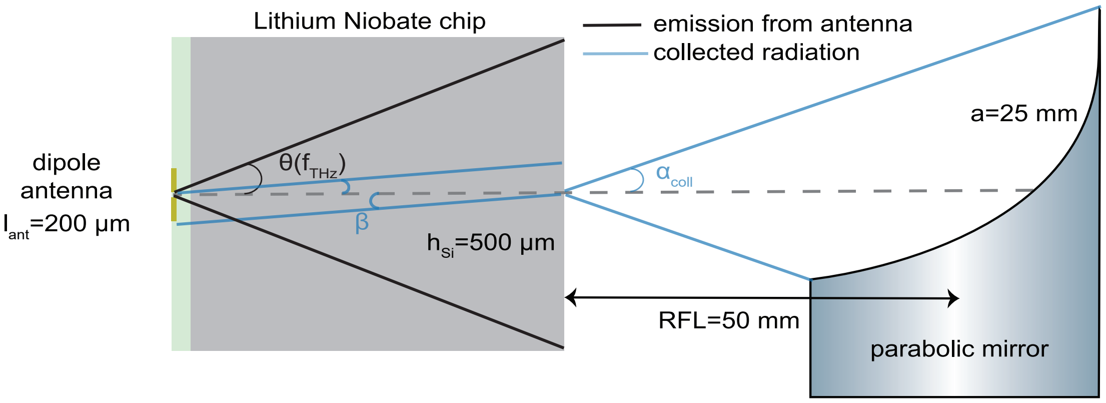

Supplementary Fig. 16: Collection of the emitted THz radiation by the parabolic mirror. The dipole antenna emits into the Silicon substrate at a frequency-dependent angle $\theta(\mathrm{f_{THz}})$. Due to diffraction from Silicon to air and due to the limited area of the parabolic mirror, only radiations at angle β can be collected. α_{coll} is the the collection angle of the parabolic mirror, RFL: reflective focal length.

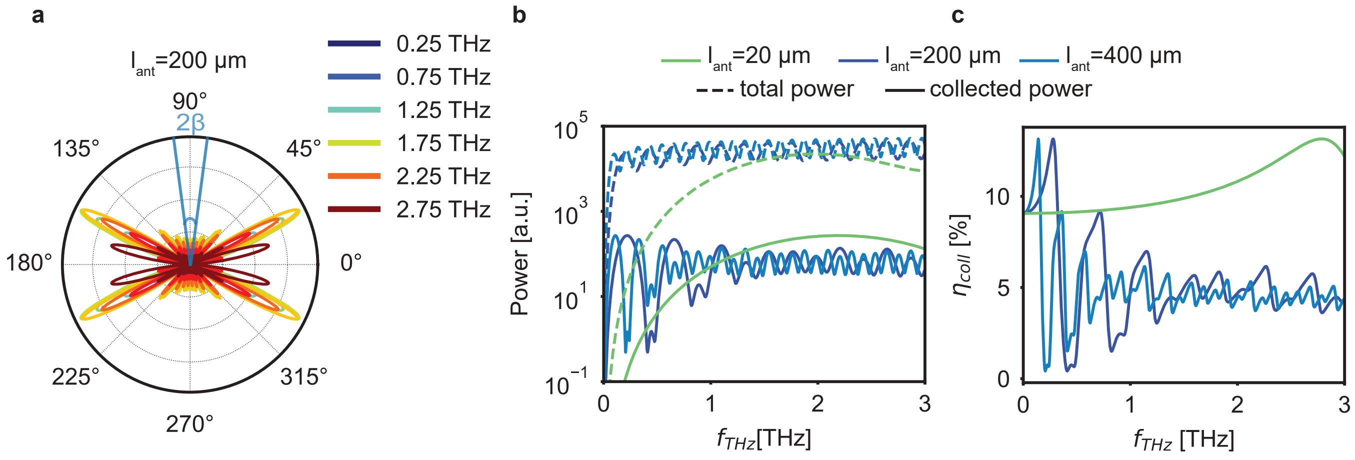

Supplementary Fig. 17: Farfield radiation patterns of our dipole antenna and collected power from the first parabolic mirror.a radiation pattern of a dipole antenna for different THz frequencies. The blue line is the collection angle inside the substrate which is constrained by the refractive index of the substrate. **b** the total and collected power values using equations 41 and 39 for different antenna length. **c** collection efficiency of the various antennas using equation 42.

where a is the radius and RFL is the reflective focal length of the parabolic mirror. In our case the angle is $\alpha_{\mathrm{coll}} = 26.6$ degrees. The corresponding angle in the substrate β is $\beta = \arcsin\left(\frac{\sin\alpha_{\mathrm{coll}}}{\mathrm{n_{Si}}}\right) = 7.55$ degrees. In Supplementary Fig. 17 a, the farfield radiation pattern is plotted using equation 38 for different frequencies and for our antenna $\mathrm{l_{ant}} = 200\ \mu$m. Next, the intensity is integrated over the collection angle

$$\mathrm{P_{collected}}(\mathrm{f_{THz}}) = \int_{\pi/2-\beta}^{\pi/2+\beta}\int_{-\beta}^{\beta} \mathrm{U}(\theta, \mathrm{f_{THz}})\mathrm{sin}\theta \mathrm{d}\phi \mathrm{d}\theta \tag{41}$$

The results of equations 41 and 39 are shown in Fig. 17 b for three different antenna lengths. While the total radiated power is similar for different antenna lengths, collection of the radiation strongly depends on the radiation pattern. It is evident that our antenna provides a flat response at frequencies above 1 THz compared to shorter antennas despite the overall decrease in collected power after reaching the resonance. The collection efficiency of the emitted electric field can be written as

$$\eta_{\mathrm{coll}} = \sqrt{\frac{\mathrm{P_{collected}}}{\mathrm{P_{total}}}} \tag{42}$$

We find that $\eta_{\mathrm{coll}} \sim 0.05$. To show that the THz beam is illuminating the parabolic mirror completely, a knife edge

measurement is conducted with 1 mm steps on a device with antenna length $l_{ant} = 200\ \mu$m. The blocking surface is placed in the collimating path after the first parabolic mirror. As clearly seen in Supplementary Fig. 18, a change in amplitude is evident along the complete range of the parabolic mirror.

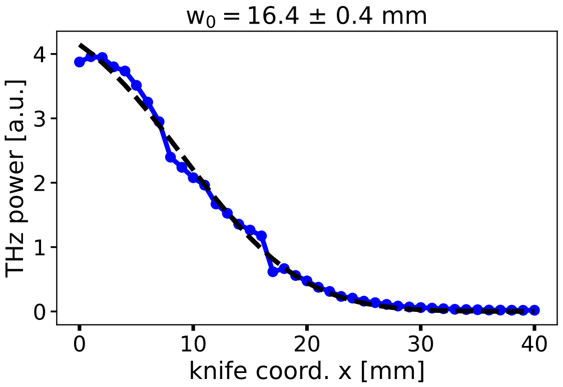

Supplementary Fig. 18: knife edge measurement of the collimated THz beam emitted from the dipole antenna in Fig. 2. Dashed line is the fitting of the power to the erf function.

3 Further supporting measurements of photonics-integrated TFLN emitters

A Estimation of collection and detection efficiencies

The emitted THz electric field from the antenna will encounter the following amplitude reflection coefficients:

$$r_{total} = r_{Si} \cdot r_{crystal} \tag{43}$$

r_{Si} is the reflection of THz at the Silicon substrate air interface. Neglecting dispersion and assuming perpendicular incidence, its absolute value is 0.54. For the detection crystals, $r_{crystal}$ amounts to 0.54 for ZnTe. The total reflection is in the orders of 0.29 and 0.28 for the crystals accordingly. One further contribution for the undetected THz is the mismatch between the mode area inside the transmission line and the area of the focused THz beam inside the crystal. Our simulations in Sec 2 A give a mode area of $S_{eff,THz} = 6\ \mu m^2$ for the measured devices with gap of 3.3 μm. The waist of the beam at the facet of the detection crystal can be calculated from

$$2w_{crystal} = \frac{4 \cdot c}{f_{THz} \cdot \pi} \cdot \frac{RFL}{2w_0} \tag{44}$$

and yields with RFL = 50 mm and $2w_0 = 32$ mm a beam waist at the crystal facet of $2w_{crystal} = 596\ \mu m$, corresponding to an area of $A_2 = \pi/2 \cdot w_{crystal}^2 = 139 \cdot 10^3 \mu m^2$ at 1 THz. The Area mismatch is given as

$$A = \frac{S_{eff,THz}}{A_2} \tag{45}$$

and at 1 THz, it amounts to $4.3 \cdot 10^{-5}$. As derived in A and shown in Supplementary Fig. 11, we estimate a peak THz amplitude in the order of $600 \cdot 10^3$V/m at the end of the transmission.

The detected THz field can be written as

$$E_{det} = E_{THz} \cdot \eta_{coll} \cdot r_{total} \cdot \sqrt{A} \tag{46}$$

where E_{THz} is the electric field in time domain derived in equation 23. Collection of the THz radiation by the optics and the antenna's farfield characteristics are included in η_{coll} equation 42. The reflection r_{total} is described in equation 43 and the area mismatch in equation 45. We estimate the terahertz electric field detected in the farfield to be approximately $\eta_{coll} \cdot r_{total} \cdot \sqrt{A} = 0.05 \cdot 0.28 \cdot \sqrt{4.3 \cdot 10^{-5}} \approx 10^{-4}$ lower than the one at the end of the transmission line. Given that for pump pulse energies of 1 pJ we estimate field amplitudes inside the transmission line of $100 \cdot 10^2$ V/m, corresponding to $100 \cdot 10^4$ V/m for 100 pJ pulse energies, this is consistent with our measurements with field amplitudes on the order of 100 V/m for pump pulse energies of 100 pJ.

We compute the as-measured efficiency of terahertz generation from our results with a mounted silicon lens in Supplementary Sec. 6D using the formula $\eta = \frac{J_{THz}}{J_{opt}}$, with $J_{opt} = 60$ pJ and $J_{THz} = \frac{1}{2} c_0 n \epsilon_0 E_{THz}^2 A_2 \tau_{THz}$ where $E_{THz} = 68.5$ V/m, $\tau_{THz} = 800$ fs is the terahertz pulse length and find $J_{THz} = 2.3 \cdot 10^{-18}$ J, which corresponds to an average power of $P_{THz} = J_{THz} \cdot f_{rep} = 0.2$ nW . The as-measured generation efficiency is about $\eta_{as-measured} = 3.9 \cdot 10^{-8}$. We can estimate the actual generation efficiency inside the transmission line $\eta_{TL,estimated} = \frac{\eta_{as-measured}}{\eta_{coll}^2 \cdot r_{crystal}^2} = 5.4 \cdot 10^{-5}$.

B Shot-noise limited detection using electro-optic sampling

In order to show that the detection scheme approaches the shot-noise limit at high modulation frequency, the fundamental limit is calculated from the experimental conditions using the equation

$$\delta V_{shot} = \sqrt{4eV_0 \frac{G}{\Re} \Delta f} \tag{47}$$

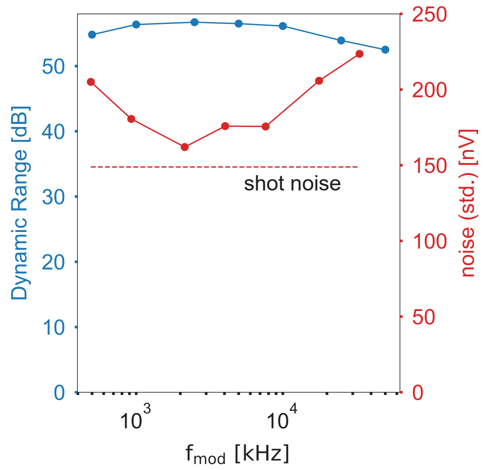

Supplementary Fig. 19: Noise and dynamic range with modulation frequency. Dashed line describes the shot noise contributions

where V_0 is the voltage on the photodetector, e is the electron's charge, G is the conversion gain in units of V/W, $\Re$ is the responsivity in units of A/W, and Δf is the detection bandwidth, it can be calculated from the chosen time constant for lock-in detection and the filter-order. Note, that additional factor of 2 comes from the assumption, that the shot noise from both photodetectors is not correlated. The noise calculations using the formula above and the parameters are summarized in the Supplementary Table 5.

G	$\Re$,	V_0	Δf	δV_{shot}
$16 \cdot 10^3$ V/W	0.5 A/W	8 V	0.135 Hz	148 nV

Supplementary Table 5: Parameters used to estimate the theoretical shot noise amplitude and comparison with experimental result

We compare this calculated shot noise limit to the experimental one. Towards this end we modulate the emitter at various frequencies between 75 kHz and 50 MHz as shown in Fig. 19. Thanks to overcoming the flicker noise at MHz frequencies, we approach the shot noise limit with weak dependency on the frequency. The dynamic range is determined from Fourier spectrum of the measured THz signals. First, the maximum power spectral density is determined $E_{max}(f_{THz}) = \max(|E(f_{THz})|)$. Next, the noise power is determined in the frequency domain from taking the average of the power spectral density before the arrival of the THz signal t_0: $N =< |E(f_{THz})|^2 >; t < t_0$. Finally, the dynamic range is calculated using the formula $DR = E^2_{max}/N$. The results are shown in Fig. 19. We obtain dynamic range values above 50 dB up to 50 MHz modulation frequencies.

C Effect of transmission line length for terahertz generation

In the following, we experimentally demonstrate the effect of the transmission line length l_{TL} on the THz generation using the setup explained in Sec. 1 C with ZnTe crystal. We investigate the emission from three devices with same antenna length $l_{ant} = 200\ \mu m$ and different transmission lines $l_{TL} = 120\ \mu m, 500\ \mu m, 2$ mm. The results of the measurements and their peak-to-peak values are depicted in Fig. 20 a,b. In contrast to our analytical simulations in Fig. 11, the THz peak amplitude continue to increase with longer transmission lines. The spectra of the THz signals are shown in Fig. 20 c. The enhancement in emission depends strongly on frequency. We quantify this trend by calculating the ratio of the power spectral densities compared to the device with $l_{TL} = 120\ \mu m$ (Fig. 20 d). In

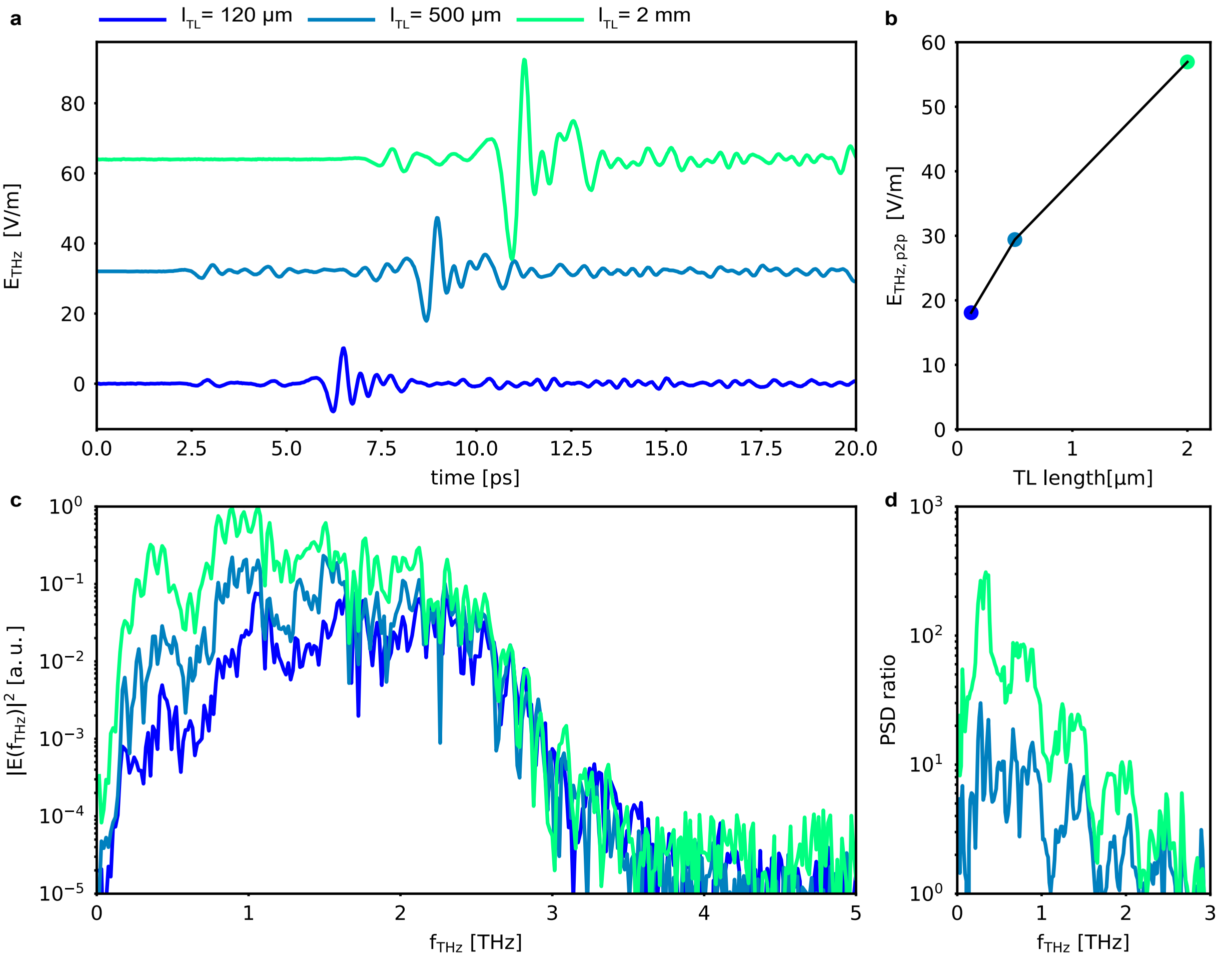

Supplementary Fig. 20: Dependence of THz emission on the length of the transmission line l_{TL} **a** detected emission from three devices with same antenna length $l_{ant} = 200\ \mu m$ and varying transmission line length $l_{TL} = 120\ \mu m, 500\ \mu m, 2\ mm$. For electro-optic sampling, ZnTe crystal is used. **b** extracted peak to peak values of the time domain traces. **c** spectra of the THz signal. **d** ratio of the power spectral densities compared to the device with $l_{TL} = 120\ \mu m$

qualitative agreement with our simulation results in Fig 11 b, lower frequencies are greatly enhanced, due to phase matching and low absorption. Frequencies above 2.2 THz are limited by phase matching and loss, limiting THz generation with longer transmission lines.

4 Theory photonics-integrated TFLN detectors

A Theory of terahertz detection inside photonics-integrated transmission lines

In this section, we derive the expressions for the detection of terahertz pulses via electro-optic sampling during the propagation of the femtosecond pulse in an optical waveguide and the co-propagation of a terahertz pulse along a transmission line. We consider the femtosecond pulses to propagate in the optical waveguide along z lab axis. Their electric field is linearly polarized (x component) and given by:

$$\mathrm{E_{pr}(x,y,z,t)} = \frac{1}{2}\mathrm{E_0}\varepsilon_{\mathrm{opt}}(\mathrm{z,t})\mathrm{g_{opt}(x,y)} \tag{48}$$

where

$$\varepsilon_{\mathrm{opt}}(\mathrm{z,t}) = \frac{1}{2\pi}\int_{-\infty}^{+\infty}\tilde{\varepsilon}_{\mathrm{opt}}(0,\omega)\mathrm{e}^{\mathrm{i}\omega(\mathrm{t}-\frac{\mathrm{z}}{\mathrm{c_0}}\mathrm{n}(\omega))}\mathrm{d}\omega \tag{49}$$

is the time domain evolution of the electric field, $\mathrm{g_{opt}(x,y)}$ is the optical mode profile, normalized such that $\iint_{(\mathrm{x,y})}|\mathrm{g_{opt}(x,y)}|^2\mathrm{dxdy}$ [illegible] where $\mathrm{S_{eff}}$ is the effective mode area and $\tilde{\varepsilon}_{\mathrm{opt}}(\omega) \equiv \tilde{\varepsilon}_{\mathrm{opt}}(0,\omega)$ the complex double-sided spectrum of the probe at the beginning of the transmission line. Through some math, we can factorize the carrier frequency of the probe ω_{pr} as follows

$$\mathrm{E_{pr}(x,y,z,t)} = \frac{1}{2}\mathrm{E_0}\varepsilon(\mathrm{z,t})\mathrm{g_{opt}(x,y)}\mathrm{e}^{\mathrm{i}(\omega_{\mathrm{pr}}\mathrm{t}-\mathrm{k_{pr}z})} + \mathrm{c.c} \tag{50}$$

where $\mathrm{k_{pr}} = 2\pi\mathrm{n_{pr}}/\lambda_{\mathrm{pr}}$ is the wave vector, $\varepsilon(\mathrm{z,t})$ is an envelope slowly varying during the propagation, and $\mathrm{E_0}$ is the probe pulse amplitude. The normalization is such, that the $\int_{-\infty}^{\infty}\iint_{(\mathrm{x,y})}1/2\varepsilon_0\mathrm{c_0n_{pr}}|\mathrm{E_{pr}(x,y,z=0,t)}|^2\mathrm{dxdydt} = \mathrm{J_{opt}}$, where $\mathrm{J_{opt}}$ is the pulse energy, and $\mathrm{c_0}$ is the speed of light in vacuum. The evolution of the probe envelope is described by:

$$\varepsilon(\mathrm{z,t}) = \frac{1}{2\pi}\int_{0}^{+\infty}\tilde{\varepsilon}(\omega_{\mathrm{THz}})\mathrm{e}^{\mathrm{i}\omega_{\mathrm{THz}}(\mathrm{t}-\frac{\mathrm{z}}{\mathrm{c_0}}\mathrm{n_g})}\mathrm{d}\omega_{\mathrm{THz}} \tag{51}$$

$\tilde{\varepsilon}(\omega_{THz}) \equiv \tilde{\varepsilon}(0,\omega_{\mathrm{THz}})$ is single-sided Fourier transform of the pulse envelope at the beginning of the transmission line, $\mathrm{n_g}$ is the group index at central probe frequency. The terahertz wave is also a wave propagating along the z-axis and delayed by a time delay τ compared to the optical pulse, hence

$$\mathrm{E_{THz}(x,y,z,t-\tau)} = \frac{1}{2}\mathrm{E_{THz}}\varepsilon_{\mathrm{THz}}(\mathrm{z,t}-\tau)\mathrm{g_{THz}(x,y)} \tag{52}$$

with similar definitions as for the optical wave but where

$$\varepsilon_{\mathrm{THz}}(\mathrm{z,t}) = \frac{1}{2\pi}\int_{-\infty}^{+\infty}\tilde{\varepsilon}_{\mathrm{THz}}(\omega_{\mathrm{THz}})\mathrm{e}^{\mathrm{i}\omega_{\mathrm{THz}}(\mathrm{t}-\frac{\mathrm{z}}{\mathrm{c_0}}(\mathrm{n_{TL}}(\omega_{\mathrm{THz}})+\mathrm{i}\kappa(\omega_{\mathrm{THz}})))}\mathrm{d}\omega_{\mathrm{THz}} \tag{53}$$

where $\tilde{\varepsilon}_{\mathrm{THz}}(\omega_{\mathrm{THz}}) \equiv \tilde{\varepsilon}_{\mathrm{THz}}(0,\omega_{\mathrm{THz}})$ the double-sided complex spectrum of the terahertz at the beginning of the transmission line. Sidebands will be generated via sum and difference frequency generation between frequency components belonging to the probe and frequency components belonging to the terahertz. The evolution of the electric field of these sidebands $\mathrm{E(x,y,z,t)}$ is described by the non-linear wave equation:

$$\left[\nabla^2 - \frac{1}{\mathrm{c_0^2}}\frac{\partial^2}{\partial\mathrm{t}^2}\right]\mathrm{E(x,y,z,t)} = \frac{1}{\varepsilon_0\mathrm{c_0^2}}\frac{\partial^2}{\partial\mathrm{t}^2}\left(\mathrm{P^L(x,y,z,t)} + \mathrm{P^{NL}(x,y,z,t)}\right) \tag{54}$$

where ε_0 is the vacuum permittivity. The linear and non-linear polarizations, $\mathrm{P^L(x,y,z,t)}$ takes the form of:

$$\mathrm{P^L(x,y,z,t)} = \varepsilon_0\int_{-\infty}^{\mathrm{t}}\chi^{(1)}(\mathrm{t}-\mathrm{t}')\mathrm{E(x,y,z,t')dt'} \tag{55}$$

and the nonlinear polarization can be computed from:

$$\begin{aligned}\mathrm{P^{NL}(x,y,z,t)} &= \varepsilon_0\chi^{(2)}(\mathrm{E_{pr}(x,y,z,t)} + \mathrm{E_{THz}(x,y,z,t}-\tau))^2 \\ &= \varepsilon_0\chi^{(2)}(\mathrm{E_{pr}(x,y,z,t)}^2 + \mathrm{E_{THz}(x,y,z,t}-\tau)^2 + 2\mathrm{E_{pr}(x,y,z,t)E_{THz}(x,y,z,t}-\tau))\end{aligned} \tag{56}$$

where $\chi^{(1)}$ is the complex linear susceptibility, and $\chi^{(2)} \approx 360\mathrm{pm/V}$ is the second-order susceptibility of $LiNbO_3$ at microwave and terahertz frequencies (below the phonon lines) [8, 9, 10]. Only the term $2\varepsilon_0\chi^{(2)}\mathrm{E_p(x,y,z,t)E_{THz}(x,y,z,t-\tau)}$ is relevant here for electro-optic sampling and it's responsible for both the up-converted and down-converted sideband. So we can rewrite

$$\left[\nabla^2 - \frac{1}{c_0^2}\frac{\partial^2}{\partial t^2}\right]\mathrm{E(x,y,z,t)} = \frac{1}{\varepsilon_0 c_0^2}\frac{\partial^2}{\partial t^2}\left(\mathrm{P^L(x,y,z,t)} + 2\varepsilon_0\chi^{(2)}\mathrm{E_{pr}(x,y,z,t)E_{THz}(x,y,z,t-\tau)}\right) \tag{57}$$

If we define $\tilde{\mathrm{E}}(\mathrm{x,y,z},\omega)$ the Fourier transform of the sideband fields and hence $\tilde{\mathrm{P}}^{\mathrm{L}}(\mathrm{x,y,z},\omega) = \varepsilon_0\chi^{(1)}(\omega)\tilde{\mathrm{E}}(\mathrm{x,y,z},\omega)$ we can now take the Fourier transform of Eq. 57 by multiplying with $\mathrm{e}^{-\mathrm{i}\omega\mathrm{t}}$ from the right and integrating over time t from $-\infty$ to $+\infty$ and find:

$$\left[\nabla^2 + \frac{\omega^2}{c_0^2}\right]\tilde{\mathrm{E}}(\mathrm{x,y,z},\omega) = \frac{1}{\varepsilon_0 c_0^2}\left(-\omega^2\varepsilon_0\chi^{(1)}(\omega)\tilde{\mathrm{E}}(\mathrm{x,y,z},\omega) + \int_{-\infty}^{+\infty}(\frac{\partial^2}{\partial t^2}\mathrm{P^{NL}(x,y,z,t)})\mathrm{e}^{-\mathrm{i}\omega\mathrm{t}}\mathrm{dt}\right) \tag{58}$$

and hence

$$\left[\nabla^2 + \frac{\omega^2(1+\chi^{(1)}(\omega))}{c_0^2}\right]\tilde{\mathrm{E}}(\mathrm{x,y,z},\omega) = -\frac{\omega^2}{\varepsilon_0 c_0^2}\int_{-\infty}^{+\infty}\mathrm{P^{NL}(x,y,z,t)}\mathrm{e}^{-\mathrm{i}\omega\mathrm{t}}\mathrm{dt} \tag{59}$$

For the moment, let's compute only the right part of the equation above:

$$\int_{-\infty}^{+\infty}\mathrm{P^{NL}(x,y,z,t)}\mathrm{e}^{-\mathrm{i}\omega\mathrm{t}}\mathrm{dt} = 2\varepsilon_0\chi^{(2)}(\frac{1}{2})^2\mathrm{E_0E_{THz}g_{opt}(x,y)g_{THz}(x,y)}(\frac{1}{2\pi})^2\int_{-\infty}^{+\infty}\varepsilon_{\mathrm{opt}}(\mathrm{z,t})\varepsilon_{\mathrm{THz}}(\mathrm{z,t}-\tau)\mathrm{e}^{-\mathrm{i}\omega\mathrm{t}}\mathrm{dt} \tag{60}$$

This integral is in fact a triple integral considering the definitions of $\varepsilon_{\mathrm{opt}}(\mathrm{z,t})$ and $\varepsilon_{\mathrm{THz}}(\mathrm{z,t})$. By solving this we find

$$\int_{-\infty}^{+\infty}\mathrm{P^{NL}(x,y,z,t)}\mathrm{e}^{-\mathrm{i}\omega\mathrm{t}}\mathrm{dt} = 2\varepsilon_0\chi^{(2)}(\frac{1}{2})^2\mathrm{E_0E_{THz}g_{opt}(x,y)g_{THz}(x,y)}\mathrm{e}^{-\mathrm{in}(\omega)\frac{\omega}{c_0}\mathrm{z}}$$
$$\cdot\frac{1}{2\pi}\int_{-\infty}^{\infty}\tilde{\varepsilon}_{\mathrm{opt}}(\omega-\omega_{\mathrm{THz}})\tilde{\varepsilon}_{\mathrm{THz}}(\omega_{\mathrm{THz}})\mathrm{e}^{\mathrm{i}(\mathrm{n_g}-\mathrm{n_{TL}}(\omega_{\mathrm{THz}}))\frac{\omega_{\mathrm{THz}}}{c_0}\mathrm{z}}\mathrm{e}^{-\kappa(\omega_{\mathrm{THz}})\frac{\omega_{\mathrm{THz}}}{c_0}\mathrm{z}}\mathrm{e}^{-\mathrm{i}\omega_{\mathrm{THz}}\tau}\mathrm{d}\omega_{\mathrm{THz}} \tag{61}$$

which tells us that at frequency ω, the total contribution to the nonlinear polarisation arises from the sum of frequency combinations $\omega - \omega_{\mathrm{THz}}$ and ω_{THz}, each weighted by their own amplitudes that are described by $\tilde{\varepsilon}_{\mathrm{opt}}(0,\omega-\omega_{\mathrm{THz}})$ and $\tilde{\varepsilon}_{\mathrm{THz}}(0,\omega_{\mathrm{THz}})$. In addition, the contribution of each pair is weighted by a term $\mathrm{e}^{\mathrm{i}(\mathrm{n_g}-\mathrm{n}(\omega_{\mathrm{THz}}))\frac{\omega_{\mathrm{THz}}}{c_0}\mathrm{z}}$ that accounts for phase matching. We can now make a few assumptions about $\tilde{\mathrm{E}}(\mathrm{x,y,z},\omega)$ that will help simplify the math. We assume its amplitude to depend on z and its spatial distribution in the xy-plane to be the same as the one of the probe, $\mathrm{g_{opt}(x,y)}$:

$$\tilde{\mathrm{E}}(\mathrm{x,y,z},\omega) = \tilde{\mathrm{E}}^{(\mathrm{amp})}(\mathrm{z},\omega)\cdot\mathrm{g_{opt}(x,y)}\mathrm{e}^{-\mathrm{ikz}}. \tag{62}$$

where $\mathrm{k} = \mathrm{n}(\omega)\frac{\omega}{c_0}$. We also multiply both sides of Eq. 59 with $\mathrm{g_{opt}(x,y)}$ and integrate over the xy-plane, so we can define an overlap function for the detection Γ_{EO} as follows:

$$\Gamma_{\mathrm{EO}} = \frac{\iint_{(\mathrm{x,y})}|\mathrm{g_{opt}(x,y)}|^2\mathrm{g_{THz}(x,y)dxdy}}{\iint_{(\mathrm{x,y})}|\mathrm{g_{opt}(x,y)}|^2\mathrm{dxdy}} \tag{63}$$

Using the slowly varying envelope approximation we find

$$[-\mathrm{i2k}\frac{\partial}{\partial \mathrm{z}} + (\frac{\omega^2}{c_0^2}(1+\chi^{(1)}(\omega)) - \mathrm{k}^2)]\tilde{\mathrm{E}}^{(\mathrm{amp})}(\mathrm{z},\omega)$$
$$= -\frac{\omega^2}{\varepsilon_0 c_0^2}2\varepsilon_0\chi^{(2)}(\frac{1}{2})^2\mathrm{E_0E_{THz}}\Gamma_{\mathrm{EO}}\frac{1}{2\pi}\int_{-\infty}^{\infty}\tilde{\varepsilon}_{\mathrm{opt}}(\omega-\omega_{\mathrm{THz}})\tilde{\varepsilon}_{\mathrm{THz}}(\omega_{\mathrm{THz}})\mathrm{e}^{-\mathrm{i}\Delta\mathrm{kz}}\mathrm{e}^{-\frac{\alpha}{2}\mathrm{z}}\mathrm{e}^{-\mathrm{i}\omega_{\mathrm{THz}}\tau}\mathrm{d}\omega_{\mathrm{THz}} \tag{64}$$

with $\Delta\mathrm{kz} = (\mathrm{n_g} - \mathrm{n_{TL}}(\omega_{\mathrm{THz}}))\frac{\omega_{\mathrm{THz}}}{c_0}\mathrm{z}$ the phase mismatch between probe and terahertz signal and $\alpha = 2\kappa(\omega_{\mathrm{THz}})\frac{\omega_{\mathrm{THz}}}{c_0}$ the frequency-dependent loss of the terahertz wave. Additionally, if $1+\chi^{(1)}(\omega) \approx \mathrm{n}(\omega)^2 - \kappa(\omega)^2 + \mathrm{i2n}(\omega)\kappa(\omega) \approx n(\omega)^2$ (losses at optical frequency are negligible $\kappa(\omega)$), we find that

$$\frac{\partial}{\partial \mathrm{z}}\tilde{\mathrm{E}}^{(\mathrm{amp})}(\mathrm{z},\omega) = \frac{\omega^2}{\mathrm{i2k}c_0^2}2\chi^{(2)}(\frac{1}{2})^2\mathrm{E_0E_{THz}}\Gamma_{\mathrm{EO}}\frac{1}{2\pi}\int_{-\infty}^{\infty}\tilde{\varepsilon}_{\mathrm{opt}}(\omega-\omega_{\mathrm{THz}})\tilde{\varepsilon}_{\mathrm{THz}}(\omega_{\mathrm{THz}})\mathrm{e}^{-\mathrm{i}\Delta\mathrm{kz}}\mathrm{e}^{-\frac{\alpha}{2}\mathrm{z}}\mathrm{e}^{-\mathrm{i}\omega_{\mathrm{THz}}\tau}\mathrm{d}\omega_{\mathrm{THz}} \tag{65}$$

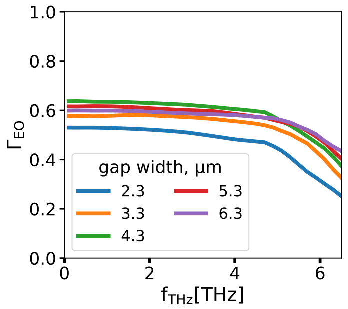

Supplementary Fig. 21: The overlap factor Γ_{EO} for the detection of various gap widths of the transmission line.

and from here we find at the end of the transmission line with length l_{TL}

$$\tilde{E}^{(amp)}(l_{TL},\omega) = -i\frac{\omega}{2c_0 n(\omega)}2\chi^{(2)}(\frac{1}{2})^2 E_0 E_{THz}\Gamma_{EO}\frac{1}{2\pi}\int_{-\infty}^{\infty}\tilde{\varepsilon}_{opt}(\omega-\omega_{THz})\tilde{\varepsilon}_{THz}(\omega_{THz})l_{TL}G_{TL}(\omega_{THz})e^{-i\omega_{THz}\tau}d\omega_{THz} \quad (66)$$

with $G_{TL}(\omega_{THz})$ the phase-matching function $G_{TL}(\omega_{THz}) = \frac{e^{-i\Delta k l_{TL}} - e^{-\alpha l_{TL}/2}}{i\Delta k l_{TL} - \alpha l_{TL}/2}$. We note that this phase matching function is identical to the one for generation. Consequently, all calculations related to the phase matching function or effective length of interaction $l_{eff}(\omega_{THz}) = l_{TL}G_{TL}(\omega_{THz})$ in Sec. 2 C are equally valid for the case of detection. The formula above is general, and describes the fact that the modulation of any optical frequency has contributions from all involved terahertz frequencies. It can be evaluated numerically for probe and terahertz pulses of arbitrary pulse durations or spectral shape, without any restrictions. This is captured by the fact that the strength of the generated optical field $\tilde{E}^{(amp)}(l_{TL},\omega)$ depends on the convolution of the optical spectrum with the terahertz spectrum.

Passing back into the time-domain, we find that after the interaction region, the new generated field is

$$E(x,y,z,t) = \frac{1}{2\pi}\int_{-\infty}^{\infty}\tilde{E}^{(amp)}(l_{TL},\omega)\cdot g_{opt}(x,y)e^{i(\omega t - kz)}d\omega \quad (67)$$

To find an analytical solution, we can make few simplifications that hold in our case. Since the optical spectrum is much broader than the terahertz spectrum (the optical probe pulse is much shorter than the terahertz pulse), $\varepsilon_{opt}(\omega-\omega_{THz}) = \varepsilon_{opt}(\omega)$ is constant over the bandwidth of the terahertz. And hence we have

$$\tilde{E}^{(amp)}(l_{TL},\omega) = -i\frac{\omega}{2c_0 n(\omega)}2\chi^{(2)}(\frac{1}{2})^2 E_0 E_{THz}\Gamma_{EO}\tilde{\varepsilon}_{opt}(\omega)\frac{1}{2\pi}\int_{-\infty}^{\infty}(\tilde{\varepsilon}_{THz}\omega_{THz})l_{TL}G_{TL}(\omega_{THz})e^{-i\omega_{THz}\tau}d\omega_{THz} \quad (68)$$

and consequently reshuffling and assuming $\omega = \omega_{pr}$ for the amplitude term only

$$E(x,y,z,t) = -i\frac{\omega_{pr}}{2c_0 n(\omega)}2\chi^{(2)}\Gamma_{EO}l_{TL}\underbrace{\frac{1}{2}E_0 g_{opt}(x,y)\frac{1}{2\pi}\int_{-\infty}^{\infty}\tilde{\varepsilon}_{opt}(\omega)e^{i(\omega t-kz)}d\omega}_{E_{pr}(x,y,z,t)}$$
$$\cdot\frac{1}{2}E_{THz}\frac{1}{2\pi}\int_{-\infty}^{\infty}\tilde{\varepsilon}_{THz}(\omega_{THz})G_{TL}(\omega_{THz})e^{-i\omega_{THz}\tau}d\omega_{THz} \quad (69)$$

and finally

$$E(x,y,z,t) = -i\frac{\omega_{pr}}{c_0 n(\omega)}\chi^{(2)}\Gamma_{EO}l_{TL}E_{pr}(x,y,z,t)\frac{1}{2}E_{THz}\frac{1}{2\pi}\int_{-\infty}^{\infty}\tilde{\varepsilon}_{THz}(\omega_{THz})G_{TL}(\omega_{THz})e^{-i\omega_{THz}\tau}d\omega_{THz} \quad (70)$$

By defining now

$$\Delta\phi(\tau) = \frac{\omega_{pr}}{c_0 n(\omega_{pr})}\chi^{(2)}\Gamma_{EO}l_{TL}\frac{1}{2}E_{THz}\frac{1}{2\pi}\int_{-\infty}^{\infty}\tilde{\varepsilon}_{THz}(\omega_{THz})G_{TL}(\omega_{THz})e^{-i\omega_{THz}\tau}d\omega_{THz} \quad (71)$$

we see that $E(x,y,z,t) = -i\Delta\phi(\tau)E_{pr}(x,y,z,t)$. From here, the total optical field is

$$E_{tot}(x,y,z,t) = E_{pr}(x,y,z,t) + E(x,y,z,t) = E_p(x,y,z,t)(1 - i\Delta\phi(\tau)) = E_p(x,y,z,t)e^{-i\Delta\phi(\tau)} \quad (72)$$

Following the same procedure as in Ref. [14] we find that for the case of one modulator in one arm,

$$\frac{\Delta \mathrm{I_{out}}(\tau)}{\mathrm{I_{out}}} = \Delta\phi(\tau) \tag{73}$$

Taking now the Fourier transform of $\Delta\phi(\tau)$ we find the power spectral density PSD:

$$\mathrm{PSD}(\omega_{\mathrm{THz}}) = |\mathcal{F}\{\frac{\Delta \mathrm{I_{out}}(\tau)}{\mathrm{I_{out}}}\}|^2 = \frac{\omega_{\mathrm{pr}}^2}{\mathrm{c}_0^2 \mathrm{n}(\omega_{\mathrm{pr}})^2}\chi^{(2)2}\mathrm{E}_{\mathrm{THz}}^2\Gamma_{\mathrm{EO}}^2\mathrm{l}_{\mathrm{TL}}^2\tilde{\varepsilon}_{\mathrm{THz}}(\omega_{\mathrm{THz}})^2\mathrm{G}_{\mathrm{TL}}(\omega_{\mathrm{THz}})^2 \tag{74}$$

We find therefore that the phase matching function $\mathrm{G_{TL}}(\omega_{\mathrm{THz}})$ contributes quadratically to the detection bandwidth. Since this function quantifies both the losses and the phase matching, we find that it is essential to achieve a low terahertz loss and a large coherence length.

5 Further supporting measurements of photonics-integrated TFLN detectors

A Effect of silicon lens on terahertz detection

When a hyper-hemispherical silicon lens is added to substrate, radiation at all angles can be collected by the antenna. To quantify this effect, we measure the device shown in Fig. 5 in the main text with and without the lens (LSH-D12-T7.13 from Batop GmbH) (Supplementary Fig. 22 a). Despite the manual alignment of the device to the center of the lens, we observe several improvements. First, we achieve more than factor 10 improvement in relative modulation of our optical probe $\Delta I/I$ (Supplementary Fig. 22 b). Second, the detected spectrum is smoother and contains less notches, which is highly beneficial for spectroscopy (Supplementary Fig. 22 c). Third, the echo of the main terahertz pulse appears considerably later around 172 ps after the main pulse instead of 12 ps as in the case of the bare chip (Supplementary Fig. 22 b). Sub-THz frequencies benefit more from implementing the lens since the divergence of the beam is inversely proportional to THz frequency and higher frequencies are more directional. The performance and the measurement configurations are shown in Supplementary Table 6. To determine the noise, we take the power spectral density of the measurement between 0 and 2.5 ps (before the arrival of the terahertz pulse). We note that despite measuring less optical power from the chip when the lens is mounted (13 μW compared to 33 μW), we measure higher noise values which we cannot explain.

	bare device	device with lens
detector	MZM structure with dipole antenna and transmission line	
detector details	$l_{ant} = 200\mu m, l_{TL} = 1mm$	
emitter	GaAs PCA (iPCA-21-05-1000-800-h from Batop GmbH)	
signal on emitter	square wave with 10 V peak to peak	
modulation frequency	15.1 kHz	
pump power	120 mW	
pulse lengths	60 fs for pump and probe	
probe setup	fiber coupled: 25 cm HNLF and 15 cm bare fiber	
power before detector	300 μW (on chip)	
power after detector	33 μW	13.7 μW
readout	photodiode	
delay line step size	25 fs	
integration time per step	100 ms	
peak modulation	0.07%	1.3%
noise power before THz pulse	-86.5 dB	-81.8 dB
noise equivalent modulation	$5 \cdot 10^{-4}$%	$1.6 \cdot 10^{-4}$%
noise equivalent field	3.7 V/m	0.6 V/m
dynamic range	60.1 dB	62.6 dB

Supplementary Table 6: setup configurations for comparing performance of our detector

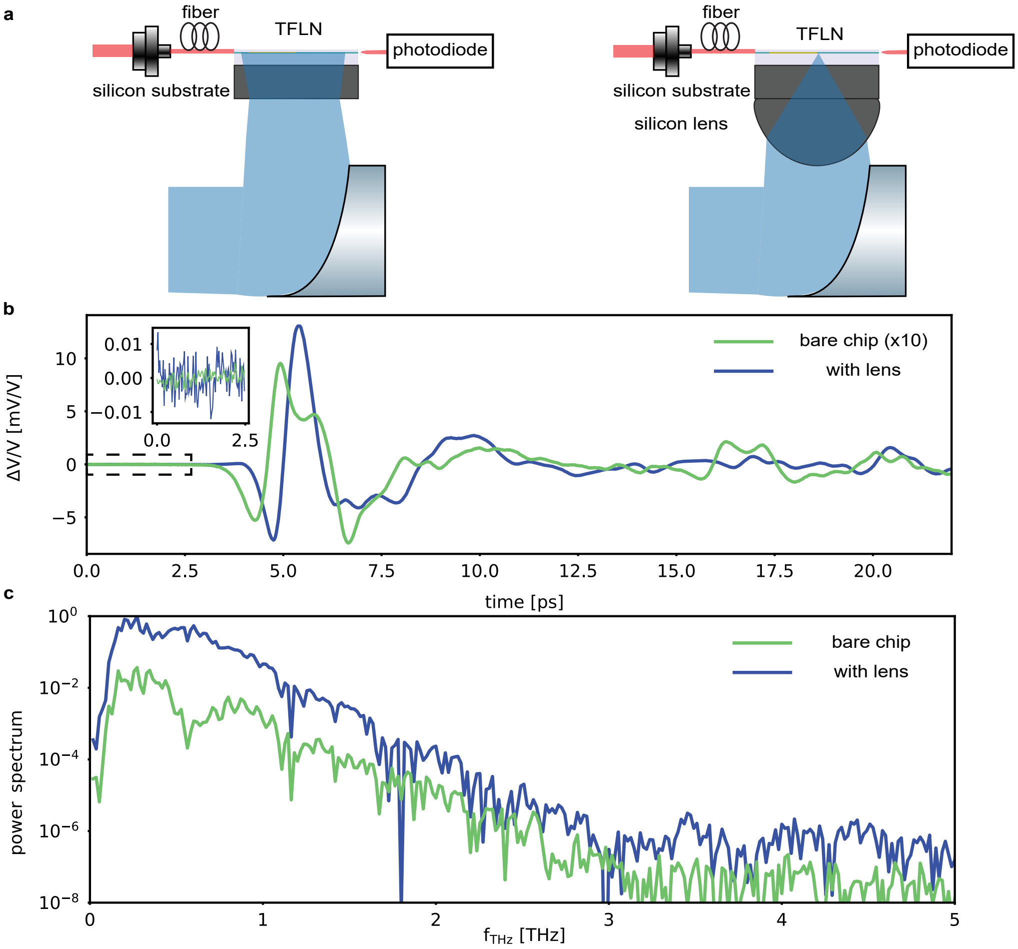

Supplementary Fig. 22: Effect of adding a silicon lens to the detector's performance a simplified sketch of the terahertz radiation for the cases of bare chip (left) and when the silicon lens is added (right). The entire setup is shown in Supplementary Fig. 26. **b** measured time traces for both cases with with $l_{ant} = 200$ μm and $l_{TL} = 1$ mm. The measurement from the bare device without a lens has been multiplied by 10 for better visibility. **c** spectra of the THz signal.

B Effect of transmission line length on terahertz detection

We now investigate the effect of the transmission line length on the efficiency of detection. For this study, we chose to not place a lens on the back-side of the chip (in contrast to Fig. 5 in the main manuscript) in order to avoid errors arising from the placement of the lens which is currently done manually and hence susceptible to misalignment. We couple our 1560 nm probe to four device with the same antenna length $l_{ant} = 200$ μm and varying transmission line lengths $l_{TL} = 125$ μm, 250 μm, 500 μm, 1 mm. For all measurements, we run a single scan with 900 ms integration time and 25 fs scan step of the delay line. As expected from equation 5 in the main text, the modulation of the probe grows linearly with l_{TL} (Fig. 23 a and b). When taking the Fourier transform, we reveal that sub-THz frequencies benefit more from longer transmission lines (Fig. 23).

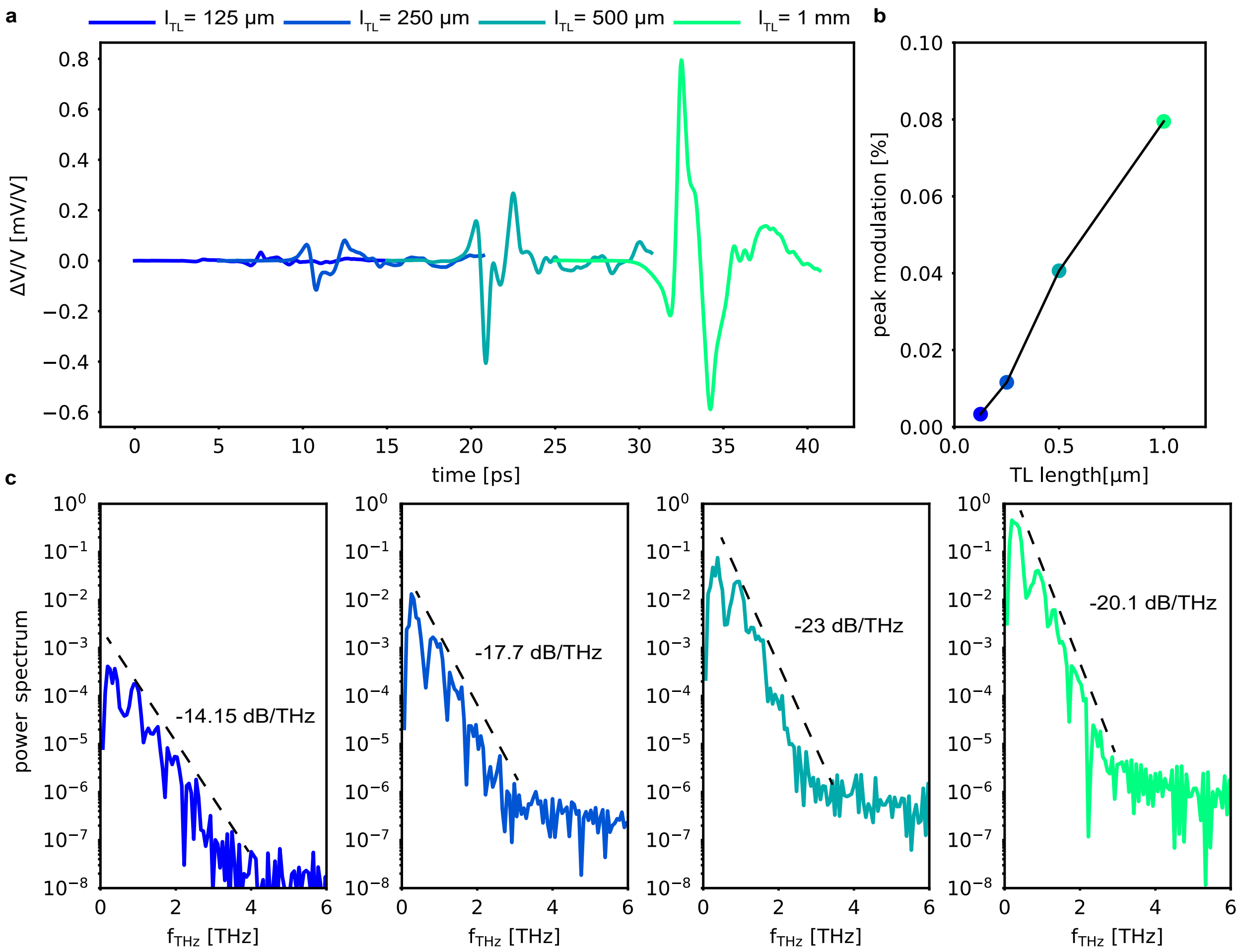

Supplementary Fig. 23: Dependence of THz detection on the length of the transmission line l_{TL}. **a** terahertz detection from four devices with the same antenna length $l_{ant} = 200\ \mu m$ and varying transmission line length $l_{TL} = 125\ \mu m, 250\ \mu m, 500\ \mu m, 1$ mm. For emission, we pump a commercial LT-GaAs at 780 nm (iPCA-21-05-1000-800-h from Batop GmbH). **b** extracted peak modulation values of the time domain traces in percentage. **c** spectra of the THz signal. Dashed lines highlight the roll-off with frequency.

6 Comparison to state of the art

A Power handling of common terahertz materials

Our work focuses on broadband terahertz generation and detection through up and down conversion of optical/terahertz pulses. While many integrated platforms are possilbe in principle, a fundamental limitation of optical power in semiconductor waveguides is the two-photon absorption due to their small bandgap compared to lithium niobate. When light propagates in a medium with two-photo absorption, the following formula can be derived from [15] to estimate the linearity of output power with input power

$$P_{out} = \frac{P_{in}}{1 + P_{in} \cdot \frac{\beta_{TPA} L}{A_{eff} \tau f_{rep}}} \tag{75}$$

where P_{in} and P_{out} are the average power values at the input and output of the waveguide, L is the propagation length, A_{eff} is the mode area, τ is the pulse length, and f_{rep} is the repetition rate. To have an intuition of the how strong is the impact on terahertz applications on chip in wide spread platforms such as InP, Si, and GaAs, we take the case of $A_{eff} = 0.45\ \mu m$ x $0.22\ \mu m$, $\tau = 60$ fs and $f_{rep} = 100$ MHz. As shown in Fig. 24, already at microwatt power values, nonlinear absorption occurs and limits the power linearity. Therefore, highly confined InP and GaAs photonic

waveguide cannot support fs pulse propagation with milliwats power values. In silicon photonics [16], average power can reach up-to few mW. This leaves TFLN as the only platform where both terahertz and optical pulses can be guided on chip with excellent power handling.

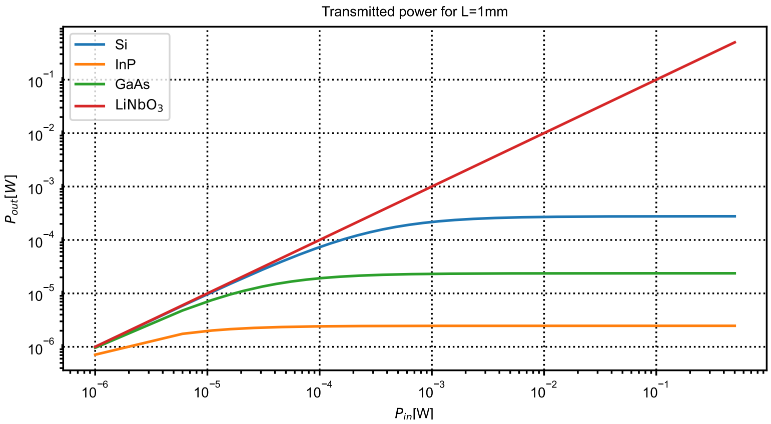

Supplementary Fig. 24: Effect of two-photon absorption (TPA) for different semiconductor materials using equation 75 which is derived from [15] We choose a waveguide with dimensions 0.45 μm x 0.22 μm and optical pump $f_{rep} = 100$ MHz,$\tau = 60$ fs. The TPA coefficients for Si, InP, and GaAs are 0.214 cm/GW [15], 24 cm/GW [17], and 2.5 cm/GW [18] accordingly. Due to the large bandgap of lithium niobate, power scales linearly in this power range.

B Benchmarking of our TFLN emitters to commercial photoconductive antenna

We place the commercial photoconductive antenna (PCA) from Batop GmbH bPCA-180-05-10-1550-h in our setup to compare its performance to our device in Fig. 2 in the main text. To improve the farfield emission from our device, a hyperhemispherical silicon lens is placed on the silicon substrate of our chip. To demonstrate the advantage in power handling, we operate at two power regimes by changing the EDFA gain and measuring the corresponding optical spectra and pulse length (Fig. 25). In the low power regime, we measure 12 mW average power in fiber before the device. This value is at the saturation limit of our PCA and the terahertz spectrum resembles the one given in the datasheet (Fig. 25 e). The PCA exceeds our device by more than factor 15 in field amplitude and 30 dB in dynamic range. When increasing the EDFA gain to give 60 mW power, spectral broadening is triggered in fiber (Fig. 25 c) and the PCA is saturated leading to degradation in the terahertz field shape. Thanks to the excellent power handling of TFLN and obtaining shorter pulses, our device provides field amplitude similar to PCA and exceeds its performance at frequencies above 0.5 THz. Compared to our device without a lens, we achieve close to factor 2 increase in field amplitude and 6 dB improvement in dynamic range. All measurement specifications are explained in Supplementary Table 7.

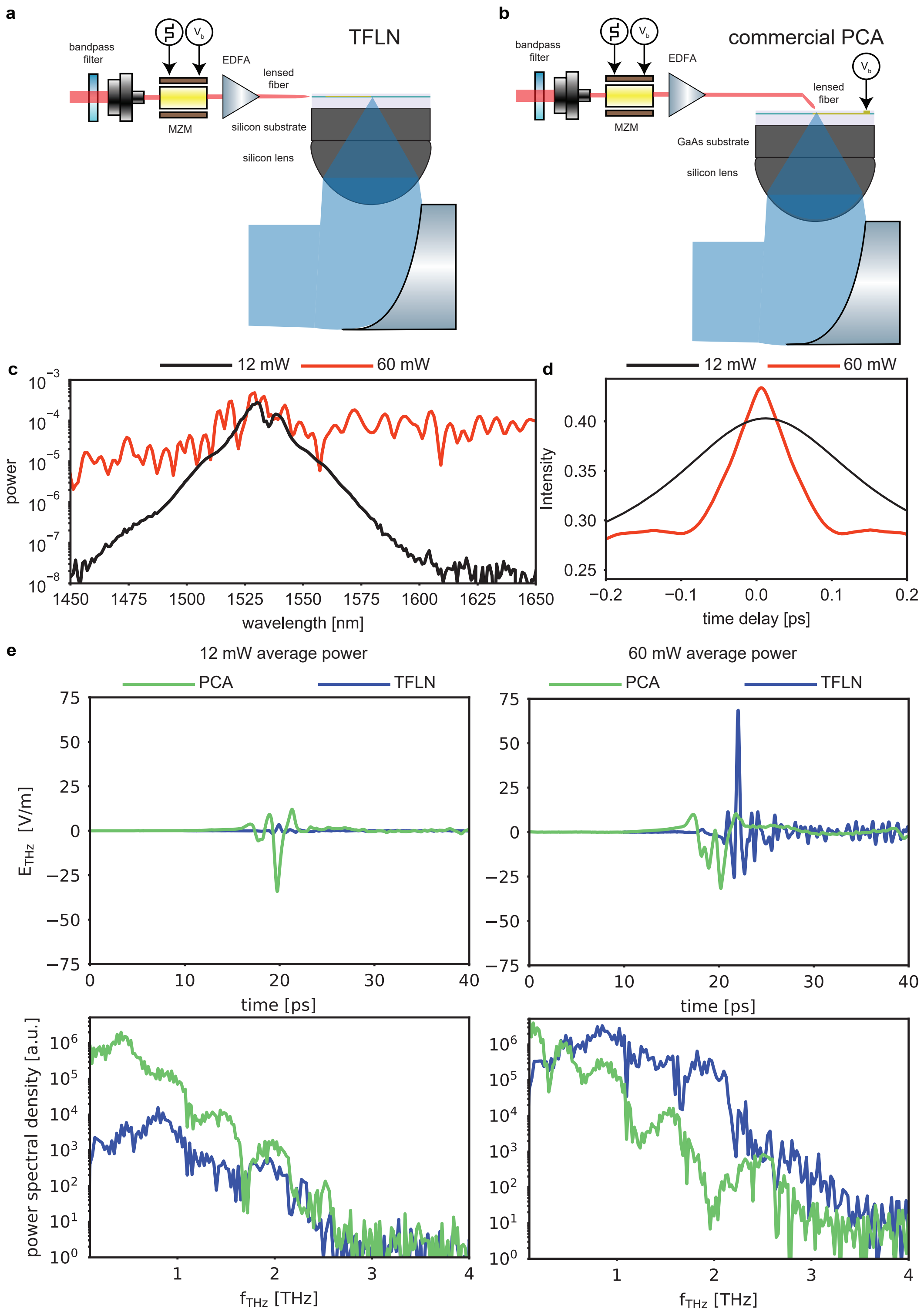

Supplementary Fig. 25: Comparison between commercial PCA emitter and our device of Fig. 2 in the main text a Simplified sketch of the optical setup for pumping the our lithium niobate terahertz emitter. A hyperhemispherical silicon lens is mounted on the backside of the chip. **b** same setup is used to pump a commercial photoconductive antenna which is biased with 10 V DC voltage. **c** Measured optical spectrum after the EDFA for 12 mW and 60 mW average power where the power is changed by changing the EDFA gain. High optical power leads to spectral broadening inside the fiber.**d** Autocorrelation measurements give 222 fs, and 63 fs for 12 mW and 60 mW accordingly.**e** measurement results from EO sampling show that our emitter can surpass commercial PCAs at higher terahertz frequencies and does not suffer from power limitation. Measurement conditions are provided in Supplementary Table 8.

C Benchmarking of our TFLN detectors to commercial photoconductive antenna

To provide a comparison with commercially available terahertz detectors, we choose a commercial PCA with similar antenna dimensions (bPCA-180-05-10-1550-h from Batop GmbH). We chose this specific model also because it also

	TFLN	commerical PCA
emitter	dipole antenna with transmission line	bowtie on InGaAs from Batop GmbH
emitter details	$l_{ant} = 200\ \mu m, l_{TL} = 2$ mm	$l_{ant} = 85\ \mu m, w_{gap} = 5\ \mu m$
bias signal on emitter	bias-free	10 V DC
pump (low power)	1.2 mW on chip	12 mW in fiber
pump (high power)	6 mW on chip	60 mW in fiber
modulation	pump modulation with 1 MHz square wave	
pump pulse length	222 fs (12 mW), 63 fs (60 mW)	
probe pulse length	58 fs	
probe setup	electro-optic sampling with 1 mm ZnTe crystal	
readout	balanced detection with 0.8 mW probe power	
delay line step size	25 fs	
integration time per step	100 ms	

Supplementary Table 7: Setup configurations for comparing different emitters.

requires low optical power, and a low applied bias voltage which is 10 V. We compare the two devices using an experimental setup as shown in Fig. 26. To retrieve the data shown in Fig. 5 of the main text, the TFLN chip is replaced by the PCA and the bare fiber is aligned to the PCA gap. All settings of the measurements are summarized for comparison in Supplementary Table 8. We see that the optical power and the integration time is lowest for our TFLN detectors. We predict that improving the in- and out-coupling from the chip could improve the achieved signal to noise ratio even further. Nevertheless, it is important to note that our chip provides integration of terahertz and optical guiding whereas the PCA does not.

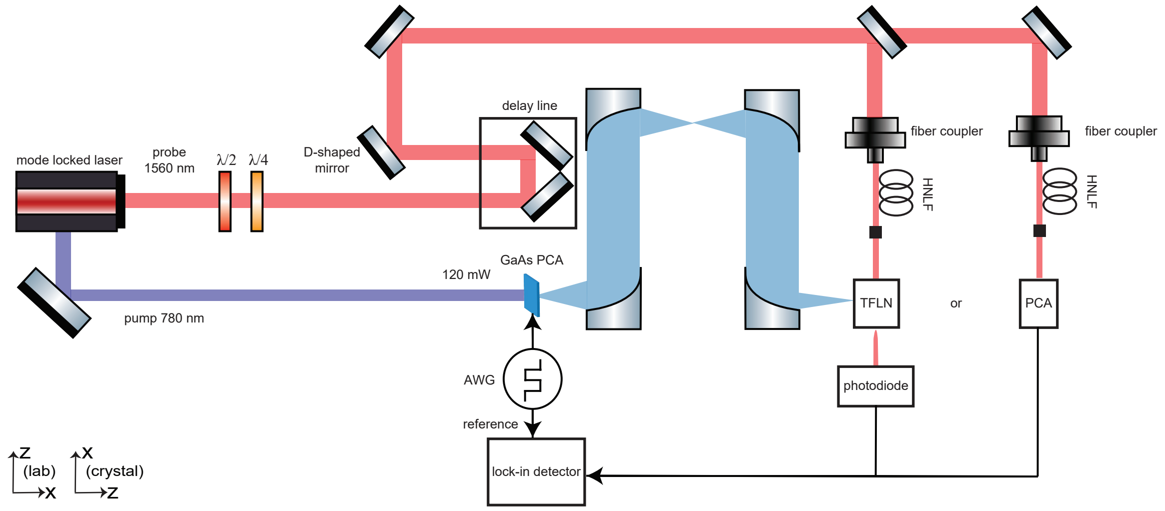

Supplementary Fig. 26: Experimental setup for THz detection in TFLN. AWG: arbitrary wave generator. HNLF: highly nonlinear fiber.

	TFLN	PCA	EOS
detector	MZM with dipole antenna	InGaAs from Batop GmbH	GaP crystal
detector details	$l_{ant} = 200\mu m, l_{TL} = 1mm$	$l_{ant} = 85\mu m, w_{gap} = 5\mu m$	L=0.2 mm r_{41}=0.87 pm/V
emitter	GaAs PCA (iPCA-21-05-1000-800-h from Batop GmbH)		
signal on emitter	square wave with 10 V peak to peak		
modulation frequency	15.1 kHz	30.1 kHz	
pump power	120 mW		
pulse lengths	60 fs for pump and probe		
probe setup	fiber coupled: 25 cm HNLF and 15 cm bare fiber		free-space
power before detector	300 μW on chip	10 mW in fiber	5 mW at crystal
power after detector	13.7 μW	absorbed	1 mW
readout	photodiode	photocurrent	balanced detection
delay line step size	25 fs	50 fs	25 fs
integration time per step	100 ms	300 ms	300 ms

Supplementary Table 8: Setup configurations for comparing different detectors.

D Overview of key metrics and properties for integrated hybrid terahertz devices

Our focus is on integrated terahertz photonic components that operate in the telecom C-band, which has motivated the development of a large variety of integrated components owing to its relevance for communication applications. We foresee these components to benefit terahertz applications as well. We compare three different integrated photonic platforms that are transparent in this band and were shown to support terahertz generation and/or detection (Supplementary Table 9). We provide an overview of their capabilities and limitations by discussing the material system itself, on-chip maximal power (which has been explained in Sec. 6 A), demonstrated THz and optical capabilities, terahertz bandwidth, and generation efficiency. In our work, we demonstrate the basic terahertz elements in thin film lithium niobate integrated circuits. In silicon photonics, guiding and detecting terahertz radiation has been previously demonstrated [16] but broadband generation through optical rectification has not yet been demonstrated in the same platform. This is likely due to the low optical powers supported by the organic material on-chip in the pulsed regime, limited to a few microwatts. III-V platforms have gained significant attention, because they integrate lasers [19, 20], modulators [21] voltage-controlled amplifiers [22] and photodiodes [23, 24] with the photonic circuit. Photonic integrated demonstrations focus mainly on continuous-wave applications, such as fully integrated generation and detection at 100-500 GHz using GaAs/AlGaAs quantum wells [25]. Pulsed terahertz applications in this platform are probably hindered by two-photon absorption of III-V semiconductors, limiting the supported optical average power to a few milliwatts for silicon [15] and sub-milliwatts for indium phosphide [17] (discussion is provided in Sec. 6 A).

All platforms have established optical capabilities in the CW regime such as the possibility to implement modulators on-chip or to realise on-chip cavities that enhance the circulating power but lithium niobate has the advantage of high power handling which extends its operation to the pulsed regime. In our generation results (Fig. 2 main text), optical rectification and proper pump conditioning lead to a frequency response of 2.25 THz 10-dB bandwidth which exceeds other available platforms. Our platform still suffer from limitations in conversion efficiency compared to photoconductive antennas [26], which could be improved by optimizing the antenna design, phase matching, optical

coupling to the waveguide, and collection of the terahertz radiation. Finally, our TFLN chips could be integrated with lasers in the visible range, which not possible with the other materials

	$LiNbO_3$	Silicon Organic	GaAs/InP
Scheme	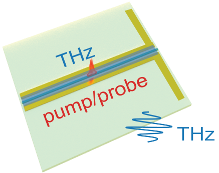	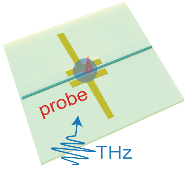	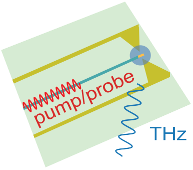
Optical pumping	CW/pulsed	CW/pulsed	CW
On-chip powers (pulsed)	more than 360 mW [27]	few mW [15]	few tens of μW [17, 18]
THz capabilities	generation[*], detection[*][28], waveguide[*], cavity [*]	detection [16], waveguide [16]	generation, detection, waveguide [29, 30], cavity [31]
Optical capabilities	modulators, high Q cavities, filter, isolators [32]	modulators, high Q cavities, filters, detectors [33]	modulators [21], lasers [19, 20], detectors [23, 24]
Bandwidth	2.25 THz (10 dB), 3.5 THz (noise floor) [*]	1 THz (10 dB), 2 THz (noise floor) [16]	0.5 THz (10 dB), 4.5 THz (noise floor) [34]
Achievable THz power	0.2 nW (estimated) [*]	generation not demonstrated	1 mW (fiber-coupled)[35], 337 nW (waveguide-integrated)[36]
Generation efficiency	10^{-7} [*]	generation not demonstrated	10^{-2} (fiber-coupled) [35]
Optical trans-parency	350 nm to 5 μm	above 1107 nm for Silicon	above 870 nm for GaAs above 925 nm for InP

Supplementary Table 9: Integrated hybrid optical-terahertz platforms. CW: countinous wave, [*]: this work

Supplementary References

[1] D. E. Zelmon, D. L. Small, and D. Jundt. "Infrared corrected Sellmeier coefficients for congruently grown lithium niobate and 5 mol.% magnesium oxide–doped lithium niobate." J. Opt. Soc. Am. B, **14**(12):3319–3322 (1997).

[2] B. N. Carnio and A. Y. Elezzabi. "Investigation of ultra-broadband terahertz generation from sub-wavelength lithium niobate waveguides excited by few-cycle femtosecond laser pulses." Opt. Express, **25**(17):20573–20583 (2017).

[3] Y.-S. Lee. Principles of terahertz science and technology, volume 170. Springer Science & Business Media (2009).

[4] S. Rajabali and I.-C. Benea-Chelmus. "Present and future of terahertz integrated photonic devices." APL Photonics, **8**(8):080901 (2023).

[5] P. C. M. Planken, H.-K. Nienhuys, H. J. Bakker, and T. Wenckebach. "Measurement and calculation of the orientation dependence of terahertz pulse detection in ZnTe." Journal of the Optical Society of America B, **18**(3):313 (2001).

[6] S. Casalbuoni, H. Schlarb, B. Schmidt, P. Schmüser, B. Steffen, and A. Winter. "Numerical studies on the electro-optic detection of femtosecond electron bunches." Phys. Rev. ST Accel. Beams, **11**:072802 (2008).

[7] Y. J. Ding. "Quasi-single-cycle terahertz pulses based on broadband-phase-matched difference-frequency generation in second-order nonlinear medium: high output powers and conversion efficiencies." IEEE Journal of selected topics in quantum electronics, **10**(5):1171–1179 (2004).

[8] G. Boyd, T. Bridges, M. Pollack, and E. Turner. "Microwave nonlinear susceptibilities due to electronic and ionic anharmonicities in acentric crystals." Physical Review Letters, **26**(7):387 (1971).

[9] G. Boyd and M. Pollack. "Microwave nonlinearities in anisotropic dielectrics and their relation to optical and electro-optical nonlinearities." Physical Review B, **7**(12):5345 (1973).

[10] B. Carnio and A. Elezzabi. "Investigation of ultra-broadband terahertz generation from sub-wavelength lithium niobate waveguides excited by few-cycle femtosecond laser pulses." Optics Express, **25**(17):20573–20583 (2017).

[11] J. Lu, J. B. Surya, X. Liu, Y. Xu, and H. X. Tang. "Octave-spanning supercontinuum generation in nanoscale lithium niobate waveguides." Optics letters, **44**(6):1492–1495 (2019).

[12] L. Xu, C. Spielmann, A. Poppe, T. Brabec, F. Krausz, and T. W. Hänsch. "Route to phase control of ultrashort light pulses." Opt. Lett., **21**(24):2008–2010 (1996).

[13] C. A. Balanis. Antenna theory: analysis and design. John wiley & sons (2016).

[14] I.-C. Benea-Chelmus, Y. Salamin, F. F. Settembrini, Y. Fedoryshyn, W. Heni, D. L. Elder, L. R. Dalton, J. Leuthold, and J. Faist. "Electro-Optic Interface for Ultrasensitive Intracavity Electric Field Measurements at Microwave and Terahertz Frequencies." Optica, **7**(5):498 (2020).

[15] X. Sang, E.-K. Tien, and O. Boyraz. "Applications of two photon absorption in silicon." Journal of optoelectronics and advanced materials, **11**(1):15 (2009).

[16] I.-C. Benea-Chelmus, Y. Salamin, F. F. Settembrini, Y. Fedoryshyn, W. Heni, D. L. Elder, L. R. Dalton, J. Leuthold, and J. Faist. "Electro-optic interface for ultrasensitive intracavity electric field measurements at microwave and terahertz frequencies." Optica, **7**(5):498–505 (2020).

[17] D. Vignaud, J.-F. Lampin, and F. Mollot. "Two-photon absorption in InP substrates in the 1.55 μm range." Applied physics letters, **85**(2):239–241 (2004).

[18] W. C. Hurlbut, Y.-S. Lee, K. Vodopyanov, P. Kuo, and M. Fejer. "Multiphoton absorption and nonlinear refraction of GaAs in the mid-infrared." Optics Letters, **32**(6):668–670 (2007).

[19] S. Jia, M.-C. Lo, L. Zhang, O. Ozolins, A. Udalcovs, D. Kong, X. Pang, R. Guzman, X. Yu, S. Xiao, et al. "Integrated dual-laser photonic chip for high-purity carrier generation enabling ultrafast terahertz wireless communications." Nature communications, **13**(1):1388 (2022).

[20] L. Schwenson, L. Liebermeister, F. Walter, S. Nellen, M. Schell, and R. B. Kohlhaas. "Photonic Integrated Continuous Wave Terahertz Spectrometer with 90 dB Dynamic Range and 4 THz Bandwidth." In "2024 49th International Conference on Infrared, Millimeter, and Terahertz Waves (IRMMW-THz)," pages 1–2. IEEE (2024).

[21] M. Theurer, T. Göbel, D. Stanze, U. Troppenz, F. Soares, N. Grote, and M. Schell. "Photonic-integrated circuit for continuous-wave THz generation." Optics Letters, **38**(19):3724–3726 (2013).

[22] S. Nellen, L. Schwenson, L. Liebermeister, M. Deumer, S. Lauck, M. Schell, and R. B. Kohlhaas. "Miniaturized continuous-wave terahertz spectrometer with 3.6 THz bandwidth enabled by photonic integration and microelectronics." IEEE Access (2024).

[23] M. Nickerson, B. Song, J. Brookhyser, G. Erwin, J. Kleinert, and J. Klamkin. "Gallium arsenide optical phased array photonic integrated circuit." Optics Express, **31**(17):27106–27122 (2023).

[24] P. A. Verrinder, L. Wang, J. Fridlander, F. Sang, V. Rosborough, M. Nickerson, G. Yang, M. Stephen, L. Coldren, and J. Klamkin. "Gallium arsenide photonic integrated circuit platform for tunable laser applications." IEEE Journal of Selected Topics in Quantum Electronics, **28**(1: Semiconductor Lasers):1–9 (2021).

[25] Y. Zhao, M. Jarrahi, et al. "Terahertz Photonics on a Chip: Monolithically Integrated Terahertz Optoelectronics based on Quantum Well Structures." arXiv preprint arXiv:2411.12046 (2024).

[26] S. Nellen, T. Ishibashi, A. Deninger, R. Kohlhaas, L. Liebermeister, M. Schell, and B. Globisch. "Experimental comparison of UTC-and PIN-photodiodes for continuous-wave terahertz generation." Journal of Infrared, Millimeter, and Terahertz Waves, **41**:343–354 (2020).

[27] M. Ludwig, F. Ayhan, T. M. Schmidt, T. Wildi, T. Voumard, R. Blum, Z. Ye, F. Lei, F. Wildi, F. Pepe, et al. "Ultraviolet astronomical spectrograph calibration with laser frequency combs from nanophotonic lithium niobate waveguides." Nature Communications, **15**(1):7614 (2024).

[28] A. Tomasino, A. Shams-Ansari, M. Lončar, and I.-C. Benea-Chelmus. "Large-area photonic circuits for terahertz detection and beam profiling." arXiv preprint arXiv:2410.20407 (2024).

[29] K. Yoshioka, G. Bernard, T. Wakamura, M. Hashisaka, K.-i. Sasaki, S. Sasaki, K. Watanabe, T. Taniguchi, and N. Kumada. "On-chip transfer of ultrashort graphene plasmon wave packets using terahertz electronics." Nature Electronics, **7**(7):537–544 (2024).

[30] M. Deumer, S. Nellen, S. Berrios, S. Breuer, S. Keyvaninia, L. Liebermeister, M. Schell, and R. Kohlhaas. "Advancing terahertz photomixers through the integration of photoconductive antennas with optical waveguides." APL Photonics, **10**(3) (2025).

[31] L. Smith, V. Shiran, W. Gomaa, and T. Darcie. "Characterization of a split-ring-resonator-loaded transmission line at terahertz frequencies." Optics Express, **29**(15):23282–23289 (2021).

[32] D. Zhu, L. Shao, M. Yu, R. Cheng, B. Desiatov, C. Xin, Y. Hu, J. Holzgrafe, S. Ghosh, A. Shams-Ansari, et al. "Integrated photonics on thin-film lithium niobate." Advances in Optics and Photonics, **13**(2):242–352 (2021).

[33] S. Shekhar, W. Bogaerts, L. Chrostowski, J. E. Bowers, M. Hochberg, R. Soref, and B. J. Shastri. "Roadmapping the next generation of silicon photonics." Nature Communications, **15**(1):751 (2024).

[34] M. Deumer, S. Nellen, S. Breuer, R. B. Kohlhaas, L. Schwenson, K. Wenzel, L. Liebermeister, M. Schell, and B. Globisch. "Waveguide-integrated photoconductive THz receivers." In "2022 47th International Conference on Infrared, Millimeter and Terahertz Waves (IRMMW-THz)," pages 1–2. IEEE (2022).

[35] A. Dohms, N. Vieweg, S. Breuer, T. Heßelmann, R. Herda, N. Regner, S. Keyvaninia, M. Gruner, L. Liebermeister, M. Schell, et al. "Fiber-coupled THz TDS system with mW-level THz power and up to 137 dB dynamic range." IEEE Transactions on Terahertz Science and Technology (2024).

[36] P. Chen, M. Hosseini, and A. Babakhani. "An integrated germanium-based THz impulse radiator with an optical waveguide coupled photoconductive switch in silicon." Micromachines, **10**(6):367 (2019).
